# FAM46C-mediated tumor heterogeneity predicts extramedullary metastasis and poorer survival in multiple myeloma

**DOI:** 10.18632/aging.204697

**Published:** 2023-05-06

**Authors:** Weilong Zhang, Chaoling Wu, Shuang Geng, Jing Wang, Changjian Yan, Xiannian Zhang, Jia-jia Zhang, Fan Wu, Yuhong Pang, Yuping Zhong, Jianbin Wang, Wei Fu, Xin Huang, Wenming Wang, Xiaoqing Lyu, Yanyi Huang, Hongmei Jing

**Affiliations:** 1 Department of Hematology, Biodynamic Optical Imaging Center (BIOPIC) and Lymphoma Research Center, Third Hospital, Peking University, Beijing 100084, China; 2 Beijing Advanced Innovation Center for Genomics (ICG), School of Life Sciences, Peking-Tsinghua Center for Life Sciences, Peking University, Beijing 100084, China; 3 Department of Hematology, Beijing Chaoyang Hospital West, Capital Medical University, Beijing 100054, China; 4 School of Life Sciences, Tsinghua-Peking Center for Life Sciences, Tsinghua University, Beijing 100190, China

**Keywords:** FAM46C, tumor heterogeneity, multiple myeloma, extramedullary metastasis, single cell sequencing

## Abstract

Cancers originate from a single cell according to Nowell’s theory of clonal evolution. The enrichment of the most aggressive clones has been developed and the heterogeneity arises for genomic instability and environmental selection. Multiple myeloma (MM) is a multiple relapse plasma cell cancer generated from bone marrow. Although there were accumulating researches in multiple myeloma pathogenesis, the heterogeneity remains poorly understood. The participants enrolled in this study were 4 EMP+ (EMP, Extramedullary plasmacytoma) and 2 EMP- primarily untreated MM patients. Single cell RNA sequencing and analysis were conducted for the single cell suspension, which was sorted by flow cytometry from peripheral blood mononuclear cells or bone marrow cells. In our research, the results of single cell RNA sequencing show that FAM46C determines MM tumor heterogeneity predicting extramedullary metastasis by influencing RNA stability. Further, we integrated and analyzed 2280 multiple myeloma samples from 7 independent datasets, which uncover that FAM46C mediated tumor heterogeneity predicts poorer survival in multiple myeloma.

## INTRODUCTION

Multiple myeloma (MM) is a multiple relapse plasma cell malignancy in the bone marrow, which is characterized by clonal proliferation of plasma cells in the bone marrow and vast secretion of monoclonal immunoglobulin [1, 2]. In recent years, the incidence of MM is clearly increased, which accounts for 2% of all hematological malignancies and severely affects the life quality of older individuals [3]. The median survival time of MM patients ranges from several months to more than 10 years. Therefore, the exact predicting of prognosis is crucial to patients with MM. As a method predicting prognosis of patients with MM, the study of MM staging system has developed in recent years. The Durie-Salmon staging system, proposed in 1975, was first used to stage MM patients [4]. The international staging system (ISS) was proposed by the International Myeloma Working Group (IMWG) in 2006, which stages MM by serum albumin and beta-2-microglobulin (β2-MG) levels [5]. Combined with molecular diagnostic techniques, a revised International Classification System (R-ISS) was proposed in 2015, which made a modification to the ISS system: Patients with high level of serum lactate dehydrogenase (LDH) and high risk of chromosomal abnormalities, including chromosomal 17p deletion and/or t (14; 16) and/or t (4; 14), are considered to be the ISS III [6, 7]. Nowadays, chemotherapy and autologous stem cell transplantation (ASCT) are main treatments for MM patients [8, 9]. With the development of genetic researches, more accurate medical treatments in MM prognosis prediction and therapy will be realized.

FAM46C, a member of nucleotidyltransferase superfamily [10–16], encodes an active non-canonical poly (A) polymerase which enhances mRNA stability [17]. Moreover, FAM46C is a high-frequency mutation gene with many frameshift and nonsense mutations which suggests that it may act as a tumor suppressor gene [18, 19]. The deletion of FAM46C was reported in approximately 20% of MM patients, whose progression-free survival (FPS) and overall survival (OS) were lower [20, 21]. Overexpression of FAM46C also enhances replication of some viruses, which is identified as a type I interferon-stimulated gene [22, 23]. In addition, the mutation of FAM46C gene in mouse causes anemia [24] and loss-of-function FAM46C is associated with bone abnormalities in mouse [10, 22, 25, 26].

Cancers originate from a single cell according to Nowell’s theory of clonal evolution [27]. The enrichment of the most aggressive clones has been developed and the heterogeneity arises for genomic instability and environmental selection [27]. Studies of next-generation sequencing in MM tumor cells have observed the presence of significant clonal heterogeneity [19, 28]. However, the study regarding the heterogeneity of MM remains limited. In our current study, we used single-cell sequencing to analyze the MM tumor cells and identified that FAM46C, by influencing RNA stability, meditated tumor heterogeneity to predict extramedullary metastasis and poorer survival.

## RESULTS

### Immunoglobulin genes specifically expressed in each multiple myeloma patient

**Figure 1:**
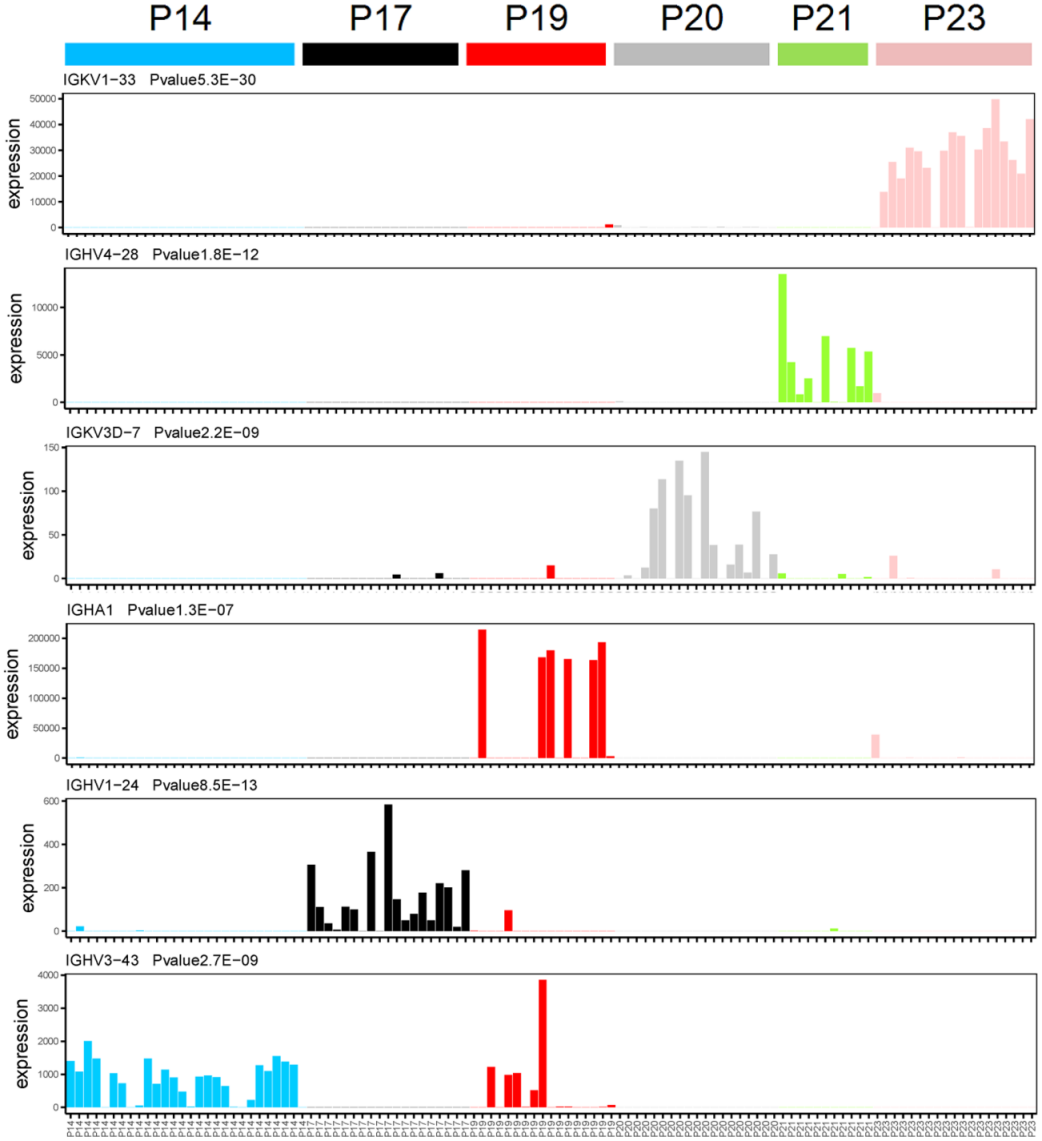
**Immunoglobulin genes specifically expressed in each patient at the single cell transcriptome level.** The X-axis and Y-axis represent the single cell and expression level (FPKM), respectively. One-way analysis of variance, the gene name and *P*-value were showed in top left corner of each figure.

In order to determine the gene expression levels of immunoglobulin in each single cell isolated from patients with MM, we performed gene expression analysis of each single cell. A total of 9 immunoglobulin genes were detected at the single cell transcriptome level, of which 6 genes were specifically expressed in different MM patients (Figure 1 and Supplementary Figure 1). Immunoglobulin genes are specifically expressed in each multiple myeloma patients at the single cell transcriptome level through one-way analysis of variance. Immunoglobulin gene IGKV1-33 is specifically expressed in P23 multiple myeloma patients (Figure 1, *P* = 5.3E-30, one-way analysis of variance), while IGHV4-28 is specifically expressed in P21 multiple myeloma patients (Figure 1, *P* = 1.8E-12, one-way analysis of variance). IGKV3D-7 and IGHA1 were specially expressed in P20 and P19 MM patients, respectively (Figure 1). Moreover, IGHV1-24 and IGHV3-43 were specially expressed in P17 and P14 MM patients, respectively (Figure 1).

Intriguingly, 3 immunoglobulin genes also were expressed in various MM patients. IGKV3D-20 and IGKV4-1 were specifically expressed in both P20 and P21 multiple myeloma patients (Supplementary Figure 1, *P* = 1.0E-24 and 5.1E-15, one-way analysis of variance). IGKC was expressed in several MM patients, including P19, P20, and P23 (Supplementary Figure 1, *P* = 2.2E-14, one-way analysis of variance). The specifically expressed immunoglobulin genes in each multiple myeloma patient with an extremely significant *P*-value suggest that we have obtained the right sample and our method of single-cell RNA sequencing is robust.

### The reduced expression of FAM46C in clonal plasma cells (CPCs) is related to RNA stability

**Figure 2:**
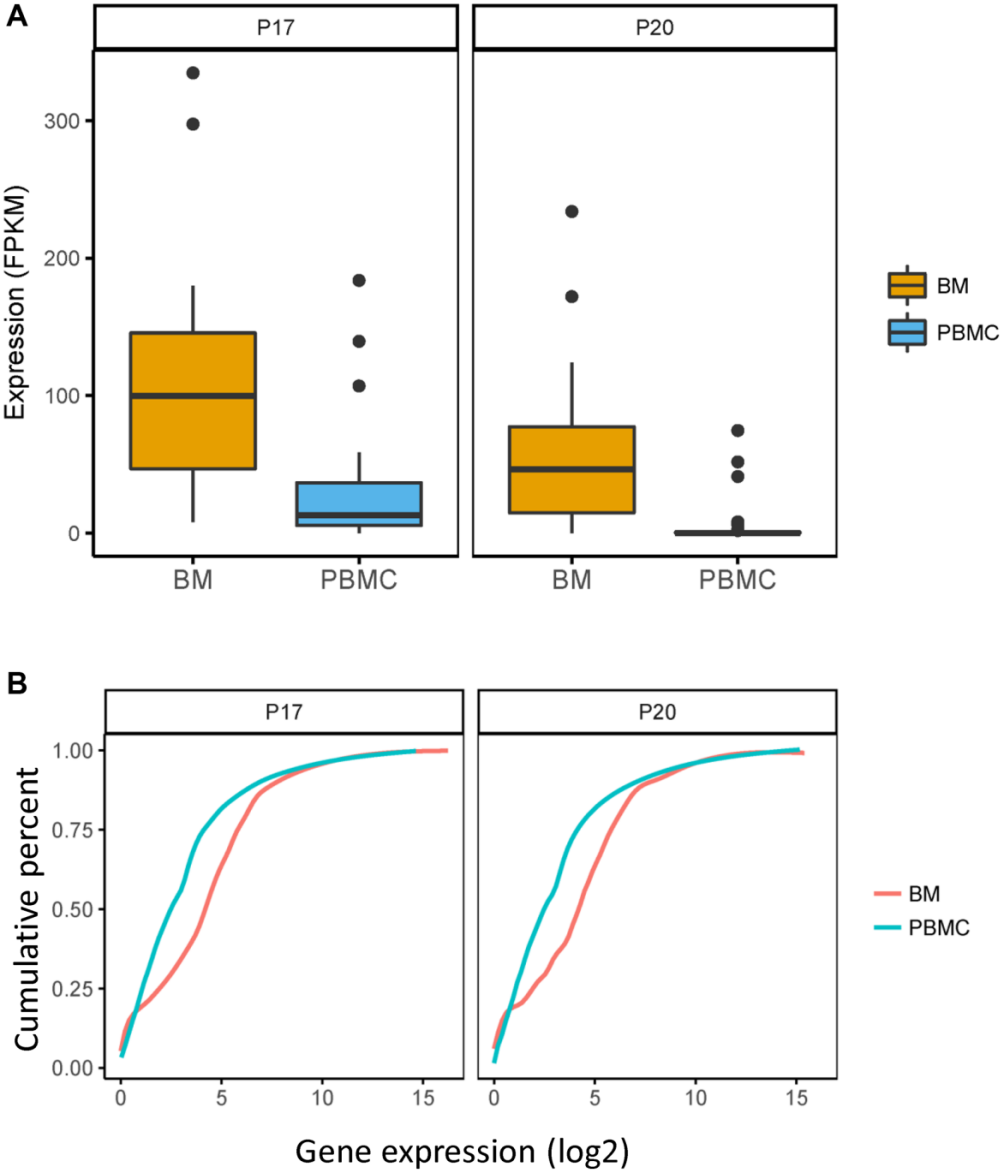
**FAM46C expressed differently in CPCs versus BMMCs in P17 and P20 patient.** Comparation of expression level of FAM46C and the whole transcriptome in CPCs and BMMCs of P17 and P20 patient. (**A**) FAM46C decreases in CPCs compared with BMMCs in P17 and P20 patients. P17: *P* = 9.4E-05, FC = −1.85; P20: *P* = 9.4E-05, FC = −3.53; Unpaired *t* test, two sided. (**B**) Lower transcriptome expression level in CPCs compared with BMMCs of single cell transcriptome in P17 and P20 patients. P17: *P* = 0.0003; P20: *P* < 0.0001; Kolmogorov-Smirnov test.

To determine the differentially expressed genes in CPCs compared with bone marrow mononuclear cells (BMMCs), we performed differential expression analysis in CPCs and BMMCs isolated from same EMP-positive patient (P17 and P20). A total of 95 genes were differentially expressed (Supplementary Table 1), of these, FAM46C was significantly lower expressed in CPCs compared with BMMCs (Figure 2A, P17: *P* = 9.4E-05, FC = −1.85; P20: *P* = 9.4E-05, FC = −3.53; Unpaired *t* test, two sided).

FAM46C acts as an active non-canonical poly (A) polymerase which enhances mRNA stability. To explore whether the decreased FAM46C level influences mRNA stability in MM, we analyzed the expression level of the whole transcriptome in both CPCs and BMMCs. As compared to BMMCs, the significantly lower level of the whole transcriptome was identified in CPCs isolated from P17 and P20 patient (Figure 2B, P17: *P* = 0.0003; P20: *P* < 0.0001; Kolmogorov-Smirnov test), indicating that decreased level of FAM46C reduces mRNA stability in MM.

### FAM46C mediated tumor heterogeneity in multiple myeloma

**Figure 3:**
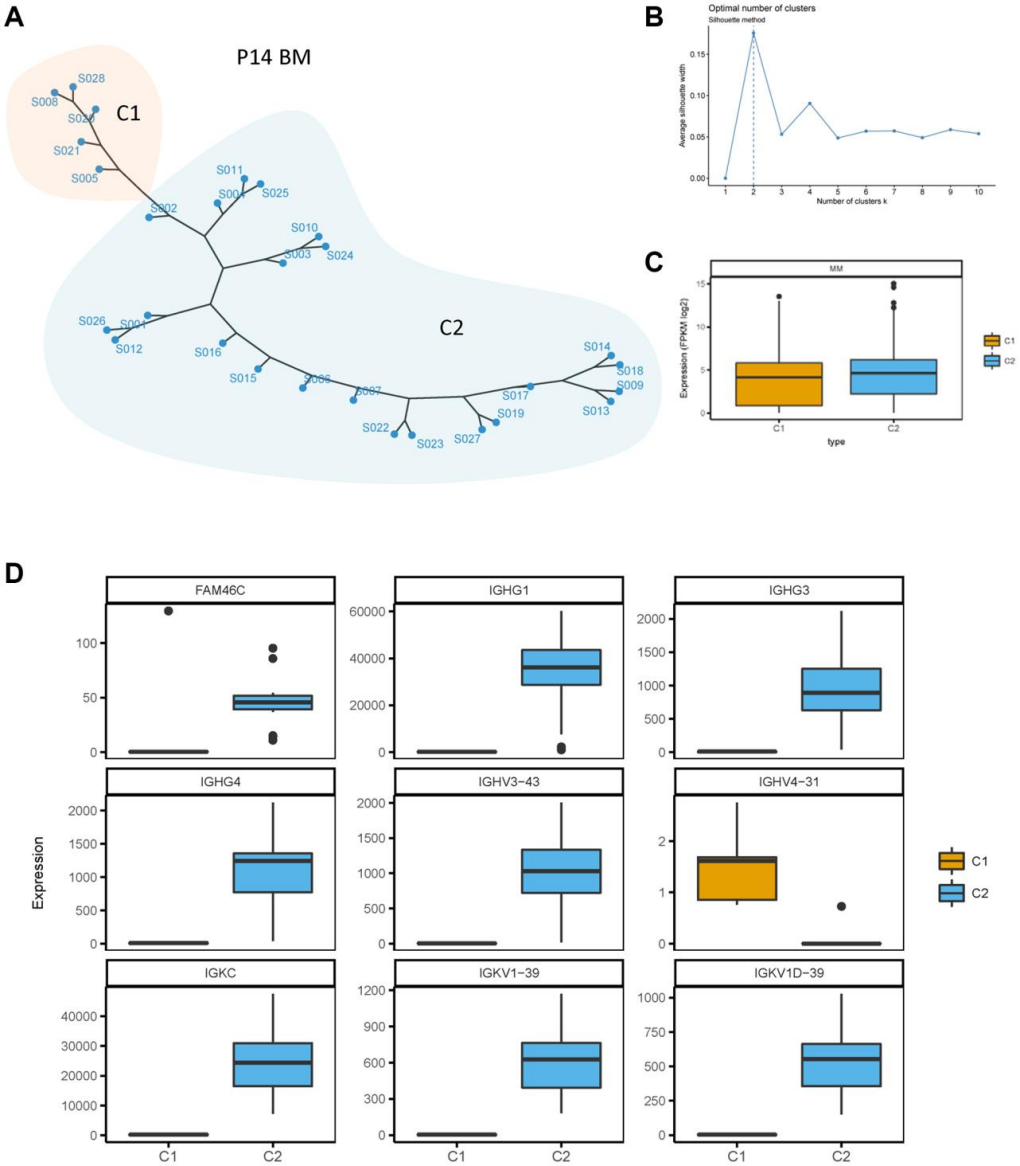
**Unsupervised clustering of BMMCs of patient P14.** (**A**) Unsupervised clustering of BMMCs of patient P14 at the single cell transcriptome level. One point means one single cell of P14 BMMCs. C1 and C2 means cluster 1 and cluster 2, showed by two different colors. (**B**) Silhouette method for cluster selecting. Average silhouette width was calculated for one to ten cluster. The cluster number was chosen with the biggest average silhouette width (dotted line). (**C**) Lower transcriptome expression level in C1 compared with C2 of single cell transcriptome in P14 patients. *P* = 0.0003, Mann Whitney test, two sided. (**D**) FAM46C and immunoglobulin genes were differently expressed between C1 and C2. *P* < 0.05, Mann Whitney test, two sided.

To further identify the heterogeneity of FAM46C in MM, we conducted an unsupervised clustering analysis for BMMCs transcriptome data of all patients. At the single-cell transcriptome level of P14 patient, two clusters (C1 and C2) of BMMCs were divided through the silhouette method according to the biggest average silhouette width (Figure 3A, 3B). The level of whole transcriptome was lower in C1 compared to C2 (Figure 3C). FAM46C was downregulated in C1 compared with C2 (Figure 3D), thereby C1 was identified as a low-FAM46C group while C2 was identified as a high-FAM46C group. Due to the decreased level of FAM46C influencing the expression of immunoglobulin genes, we analyzed the expression level of immunoglobulin genes in both low-FAM46C and high-FAM46C group. Similar to FAM46C, immunoglobulin genes were also differentially expressed between low-FAM46C and high-FAM46C group. The majority of immunoglobulin genes, such as IGHG1, IGHG3, IGHG4, IGHV3-43, IGKC, IGKV1-39, and IGKV1D-39, were downregulated in low-FAM46C group (Figure 3D). In contrast, IGHV4-31 was downregulated in high-FAM46C group.

In addition, FAM46C mediated tumor heterogeneity was also observed in BMMCs isolated from P17, P19, P20, P21 and P23 multiple myeloma patients. There were 3 clusters of BMMCs were divided in both P17 (Supplementary Figure 2A, 2B) and P19 (Supplementary Figure 3A). In P19 BMMCs, the C1 and C3 were FAM46C low group and C2 was FAM46C high group (Supplementary Figure 3B, *P* < 0.05, unpaired *t* test, two sided). Immunoglobulin gene IGLV3-1 decreased in the C1 compared with C2 in P19 BMMCs (Supplementary Figure 3B, *P* < 0.05, unpaired *t* test, two sided). In P17 BMMCs, C1 + C3 was low-FAM46C group and C2 was high-FAM46C group (Supplementary Figure 2B). In contrast to P14 BMMCs, IGKC, IGKV1-39, IGKV1D-39 showed the upregulated trend in low-FAM46C group compared with high-FAM46C group, but the difference was not significant (Supplementary Figure 2B). IGLV3-1 was significant downregulated in C1 compared with C2 (Supplementary Figure 3B).

According to silhouette method, the BMMCs of P20, P21, and P23 were also divided into 2 clusters (Supplementary Figures 4A, 4B, 5A, 5B, 6A and 6B). In P20 BMMCs, FAM46C showed elevated expression in C1 compared with C2, but the difference was not significant (Supplementary Figure 4B). However, IGLV3-19 was significantly upregulated in C1 compared with C2 (Supplementary Figure 4B). Similar to P20 BMMCs, FAM46C also has not significant difference in C1 and C2 of P23 BMMCs (Supplementary Figure 6B) while IGKV1-33 and IGKV1D-33 were significantly downregulated in C1 compared with C2 (Supplementary Figure 6B). In P21 BMMCs, the C1 was low-FAM46C group while C2 was high-FAM46C group (Supplementary Figure 5A, *P* < 0.05, unpaired *t* test, two sided). A total of 11 immunoglobulin genes were downregulated in the low-FAM46C group compared with high-FAM46C group (Supplementary Figure 5B). Immunoglobulin genes such as IGHG1, IGHG3, IGHG4, IGKC, IGKV3-11, IGKV3-20, IGKV3D-11, IGKV3D-20, IGKV4-1, IGLL5 decreased in the C1 compared with C2 in P21 BMMCs (Supplementary Figure 5B, *P* < 0.05, unpaired *t* test, two sided). In P23 BMMCs, the C1 trended to be FAM46C high group and C2 trended to be FAM46C low group (Supplementary Figure 6, *P* > 0.05 but show the trend, unpaired *t* test, two sided). Immunoglobulin genes such as IGKV1-33, IGKV1D-33 decreased in the C1 compared with C2 in P23 BMMCs (Supplementary Figure 6, *P* < 0.05, unpaired *t* test, two sided). Collectively, these results further demonstrate that FAM46C mediated tumor heterogeneity in MM.

### Single cell model of extramedullary metastasis in EMP^+^ patient

**Figure 4:**
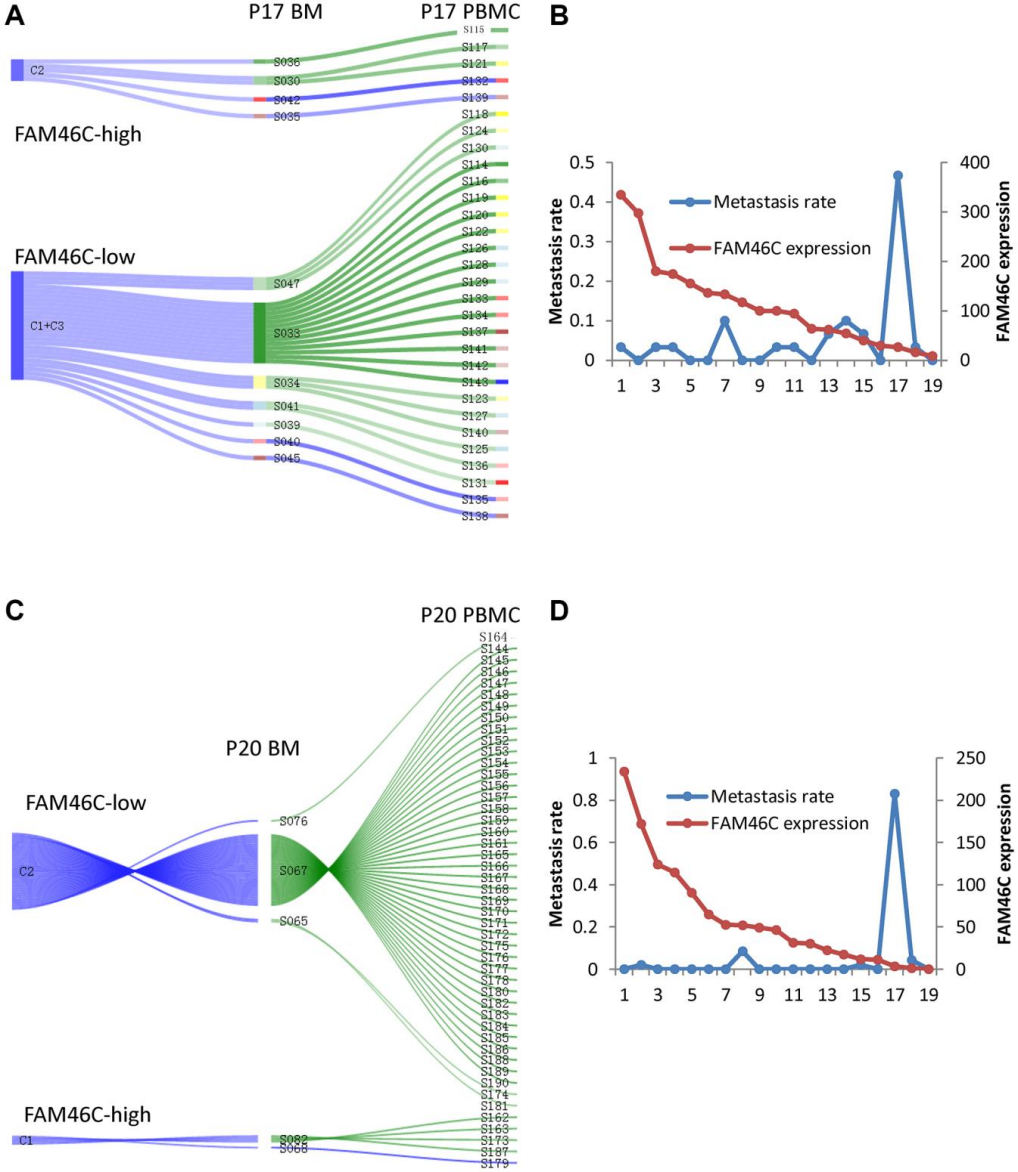
**The model of extramedullary metastasis in EMP^+^ patient P17 and P20 at single cell transcriptome level.** (**A**) Sankey diagram shows the model of extramedullary metastasis in EMP^+^ patient P17 at single cell transcriptome level. 3 clones were shown the right is all the CPCs in EMP^+^ patient P17. The middle is the related BMMCs in EMP^+^ patient P17. The left is the cluster of the BMMCs in EMP^+^ patient P17. The line between CPCs and BMMCs means that BMMC with the best Pearson correlation was chosen for a certain CPC. (**B**) The relationship between FAM46C expression level and metastasis rate in EMP^+^ patient P17. (**C**) Sankey diagram shows the model of extramedullary metastasis in EMP^+^ patient P20 at single cell transcriptome level. 2 clones were shown. The right is all the CPCs in EMP^+^ patient P20. The middle is the related BMMCs in EMP^+^ patient P20. The left is the cluster of the BMMCs in EMP^+^ patient P20. The line between CPCs and BMMCs means that BMMC with the best Pearson correlation was chosen for a certain CPC. (**D**) The relationship between FAM46C expression level and metastasis rate in EMP^+^ patient P20.

Due to having identified that FAM46C-mediated tumor heterogeneity of MM and lower expression of FAM46C in CPCs compared with BMMCs, we constructed a single sell model to show the contribution of FAM46C-meditated tumor heterogeneity for extramedullary metastasis. In P17 patient, the low-FAM46C group (C1+C3) provided 83.3% of the contribution for extramedullary metastasis, whereas the high-FAM46C group (C2) provided only 16.7% (Figure 4A). Then, we further assessed the metastasis rate (means the contribution for extramedullary metastasis) of each BMMC, the metastasis rate of the low-FAM46C BMMCs was significantly higher than high-FAM46C BMMCs (Figure 4B). Similarly, the low-FAM46C group (C2), in P20 MM patient, provided more contribution than the high-FAM46C group (C1) for extramedullary metastasis (Figure 4C), and the metastasis rate of the low-FAM46C BMMCs was also significantly higher than high-FAM46C BMMCs (Figure 4D).

### FAM46C expression is an independent prognostic factor

FAM46C mediated tumor heterogeneity contributes to extramedullary metastasis of multiple myeloma. To further explore whether FAM46C could predict poor survival of MM patients at the population level, we integrated 7 independent datasets, a total of 2280 MM samples (2072 patients), and subsequently analyzed the relationship between FAM46C gene expression and clinical characteristics of MM.

We analyzed the FAM46C expression in different ISS stages and molecular subtypes. FAM46C was lower expressed in ISS stage II compared with ISS stage I, and was no difference between ISS stage III and ISS stage II/I (Supplementary Figure 7A, *P* = 0.0078; Kruskal-Wallis test). After combining with molecular subtype, similar results were also identified in immunoglobulin G subtype of MM (Supplementary Figure 7B, *P* = 0.032; Kruskal-Wallis test). In another independent dataset with 293 multiple myeloma samples, FAM46C was lower expressed in ISS stage III compared with ISS stage I and II (Supplementary Figure 8, *P* = 0.011, *P* = 0.028; Kruskal-Wallis test). FAM46C was lower expressed in FISH 1q21 amplification samples (≥4 copies) compared with 2 and 3 copies (Supplementary Figure 9A, *P* = 0.00094, *P* = 0.009; Kruskal-Wallis test). FAM46C was lower expressed in proliferation and nuclear factor kB (NFKB) molecular subtypes (Supplementary Figures 9B and 10, *P* < 0.01, *P* < 0.001; ANOVA test).

**Figure 5:**
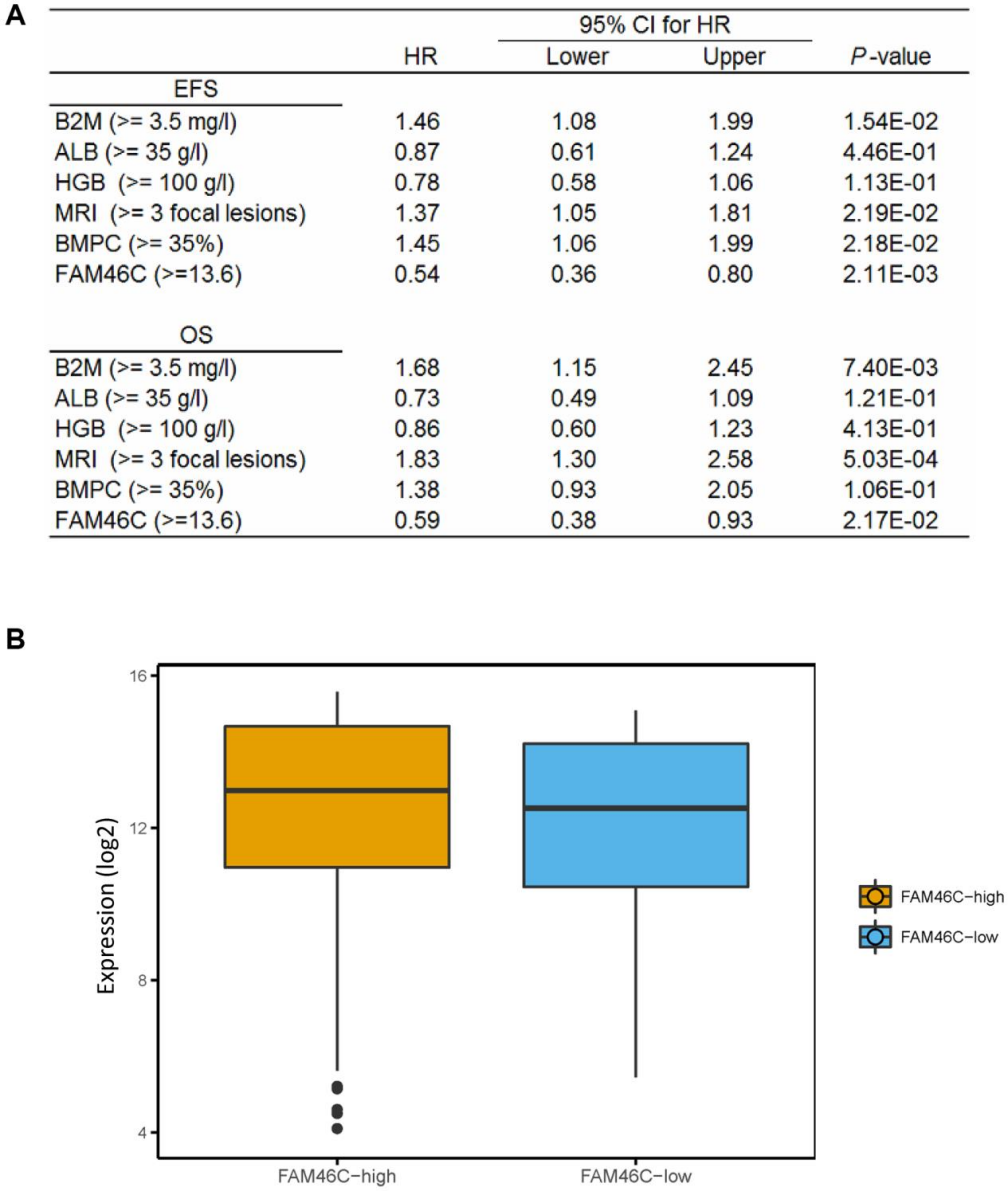
**The expression of FAM46C is an independent prognostic factor in multiple myeloma.** (**A**) Cox regression analysis identified FAM46C expression (cut off FAM46C ≥ 13.6) as an independent prognostic factor in 559 multiple myeloma patients. OS, overall survival; EPS, progress free survival. Abbreviations: β2-MG: β2 microglobulin; ALB: serum albumin; HR: hazard ratio; CI: confidence interval. (**B**) Lower transcriptome expression level in FAM46C-low group compared with FAM46C-high group in 559 multiple myeloma patients. *P* = 0.0043, unpaired *t* test, two sided (cut off FAM46C ≥ 13.6).

After the further integration of 7 independent datasets, we got a total of 559 MM samples with survival time. We analyzed the relationship between FAM46C gene expression and survival time. Multivariate Cox regression analysis showed that FAM46C was an independent prognostic factor of OS and EFS. FAM46C is a prognostic factor independent of β2-MG, HGB, ALB, MRI and BMPC (Figure 5A). For ISS stage I MM patients, FAM46C can also distinguish some patients based on poor survival (Supplementary Figure 11A, EFS, *P* = 0.0029; OS *P* = 0.016; Log-rank test). For stage II and III MM patients with lower FAM46C expression, an extremely poorer survival can be observed in these patients (Supplementary Figure 11B, EFS, *P* < 0.0001; OS *P* = 0.00025; Log-rank test). The 559 multiple myeloma patients can be divided into FAM46C-low group (57 patients) and FAM46C-high group (502 patients) (Supplementary Figures 11, 12).

We compared the whole transcriptome expression level between the low-FAM46C and the high-FAM46C group. Lower transcriptome expression level was identified in the low-FAM46C group compared with the high-FAM46C group in 559 MM patients (Figure 5B).

Moreover, we further investigated whether clinical outcomes were different between the low-FAM46C and the high-FAM46C group. For 295 ISS stage I MM patients, Kaplan-Meier survival curves indicated that patients with lower FAM46C expression had poorer clinical outcomes (shorter OS and EFS time) (Supplementary Figure 12A). Similar results were also observed in 263 ISS stage II and III MM patients (Supplementary Figure 12B).

### FAM46C were related to LDH level and clinical therapy in multiple myeloma patients

**Figure 6:**
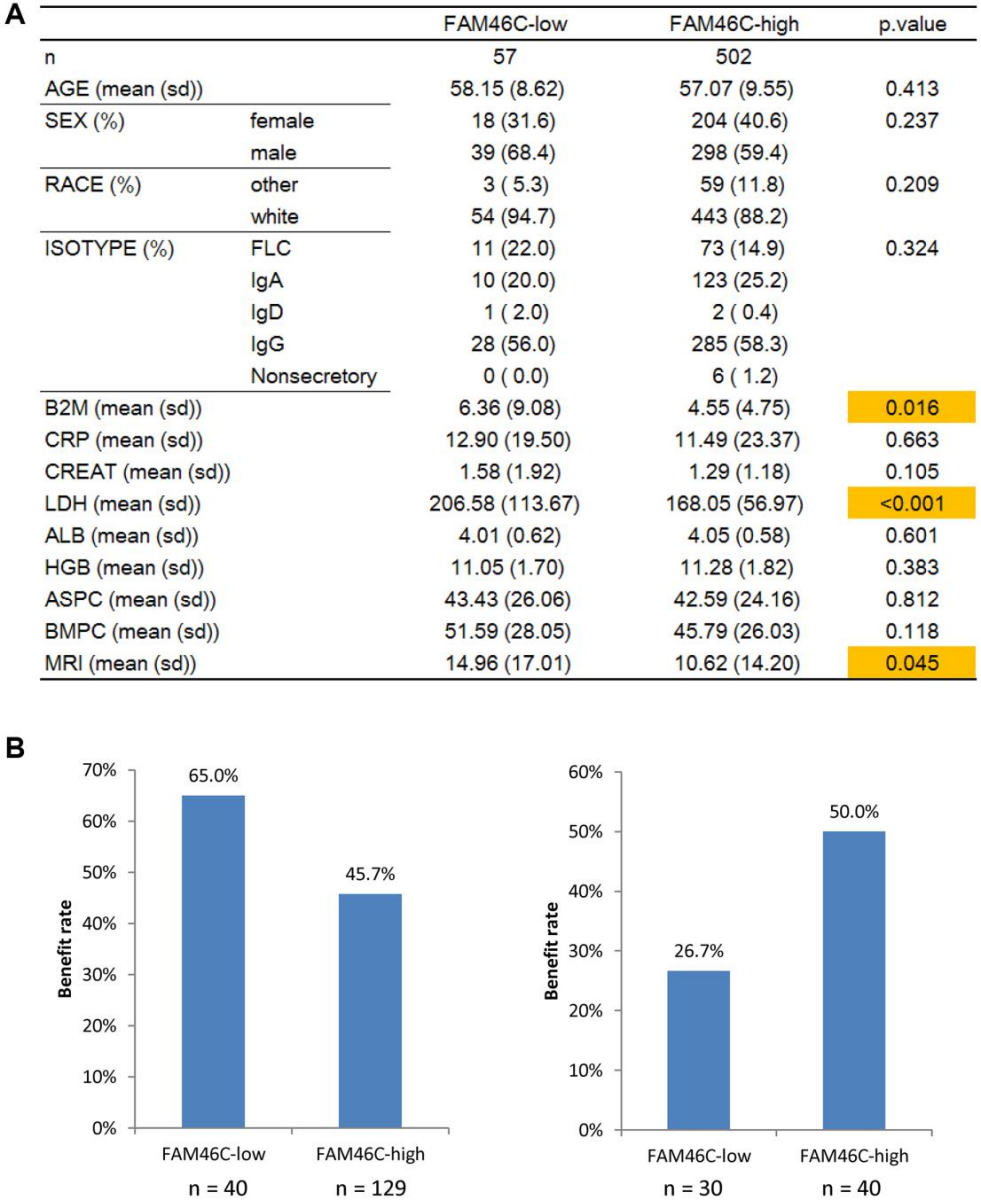
**FAM46C was related to LDH level and clinical therapy in in multiple myeloma patients.** (**A**) The comparison of clinical information between FAM46C-low and FAM46C-high group. (**B**) FAM46C was related to bortezomib and dexamethasone therapy in multiple myeloma patients. Left, bortezomib, *P* = 0.03, Chi-square test, two sided. Right, dexamethasone, *P* = 0.048, Chi-square test, two sided.

Further analysis of clinical characteristics between low-FAM46C and high-FAM46C group revealed that they were similar in age, sex, race, etc. However, several blood indexes (Figure 6A, *P* = 0.016, *P* < 0.001, *P* = 0.045, unpaired *t* test, two sided), especially LDH, were lower in high-FAM46C group. Among them, LDH is the most significantly different clinical characteristic (Figure 6A, *P* < 0.001, unpaired *t* test, two sided). LDH is active in the anaerobic glycolysis, which seems closely related to FAM46C. So, we compared the expression of hypoxia-related genes, including HIF1A, HIF1AN, and HIF3A, between low-FAM46C and high-FAM46C group, but none of them were significantly expressed (Supplementary Figure 13, *P* > 0.05, unpaired *t* test, two sided). Interestingly, 2 other hypoxia related genes (CITED2 and TAZ) were significantly expressed in FAM46C-low vs. FAM46C-high group (Supplementary Figure 13, *P* < 0.0001, unpaired *t* test, two sided). CITED2 was the top 1 down regulated gene in FAM46C-low vs. FAM46C-high group (Supplementary Figure 13, *P* < 0.0001, unpaired *t* test, two sided).

Moreover, we analyzed gene expression profiles and the clinical therapy response in another 239 multiple myeloma patients. FAM46C was related to bortezomib and dexamethasone therapy in multiple myeloma patients (Supplementary Figure 14). FAM46C was more highly expressed in MR patients with dexamethasone therapy (*P* < 0·01; *P* < 0·05; ANOVA test; Supplementary Figure 14) and in PR patients with VAD (Vincristine, Adriamycin, and Dexamethasone) therapy (*P* < 0·05; ANOVA test; Supplementary Figure 15). The low-FAM46C group harbored a much higher benefiting rate to the bortezomib treatment than the high-FAM46C group (Figure 6B). Conversely, low-FAM46C group had only a 26.7% benefiting rate to the dexamethasone treatment, which was much lower compared to the high-FAM46C group (50%) (Figure 6B).

Furthermore, we assessed the gene expression of FAM46C before and after treatment. As compared to baseline, the gene expression level of FAM46C was significantly lower in relapsed MM with TT2 treatment (autologous hematopoietic stem-cell transplantation and thalidomide therapy) or TT3 treatment (incorporating bortezomib up-front into a tandem transplant regimen) (Supplementary Figure 16). This suggested that FAM46C-mediated tumor sub clone (lower FAM46C sub clone) faced a positive selection during the tumor clonal evolution. Consistently, this clonal evolution was also observed in two other independent datasets. FAM46C is lowly expressed in relapsed MM with at least one autologous hematopoietic stem-cell transplantation (ASCT) compared with presentation MM (Supplementary Figure 17A). Moreover, a similar result was observed in relapsed MM after chemotherapy and pre-1^st^ bone marrow transplant compared with baseline MM (Supplementary Figure 17B).

### The cell proliferation capacity of H929 cell was potentiated with the knockdown of FAM46C

**Figure 7:**
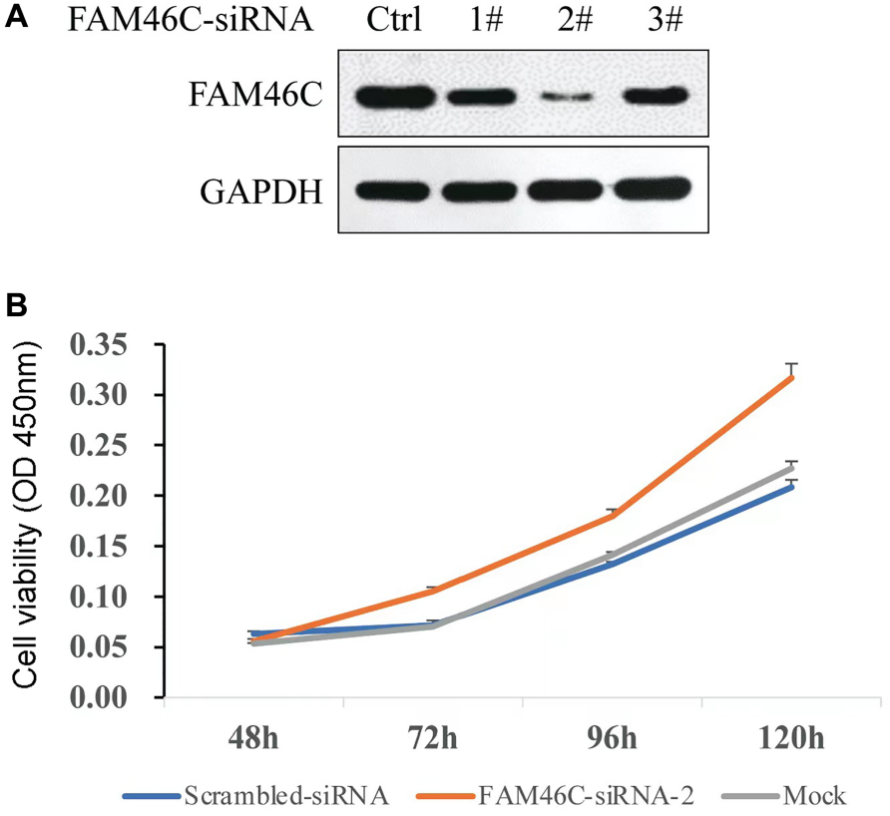
**The capacity of cell proliferation was potentiated by the reduction of FAM46C expression in H929 cell.** (**A**) The selection of FAM46C-siRNA by western-blot, Ctrl: control; 1#, 2#, and 3# represent the number of each FAM46C-siRNA. (**B**) The detection of cell proliferation capacity in H929 cells.

The best FAM46C-siRNA was selected based on the results of western blot (Figure 7A). To identify whether FAM46C could affect the proliferation capacity, FAM46C-siRNA-2 was transfected into H929 cells and Cell Counting Kit-8 (CCK8) assay was used to detect cell proliferation capacity. We found that the reduction of FAM46C expression could enhance cell proliferation capacity significantly, particularly at 96h (Figure 7B, *P* = 3.2E-4, ANOVA test) and 120 h (Figure 7B, *P* = 3.0E-4, ANOVA test).

## DISCUSSION

Cancers originate from a single cell according to Nowell’s theory of clonal evolution [27]. The enrichment of the most aggressive clones has been developed and the heterogeneity arises for genomic instability and environmental selection [27]. In our research, the results of single cell RNA sequencing show that the FAM46C-meditated tumor heterogeneity predicts extramedullary metastasis and poorer survival in multiple myeloma by influencing RNA stability.

By analyzing the single cell RNA sequencing data of BMMCs from 6 multiple myeloma patients, we found the BMMCs from 3 patients could be divided into a significantly FAM46C-low and a FAM46C-high group. The other 3 patients trended to be one of the FAM46C-low and FAM46C-high group. There are 4 points that support the heterogeneity in BMMCs: (a) Unsupervised clustering of BMMCs of patients were conducted, which supports that FAM46C is a statistically dominate factor in distinguishing between clusters of BMMCs and the heterogeneity in BMMCs. (b) The immunoglobulin genes which are known as the hallmarks of clonal plasma cells were differently expressed among clusters of BMMCs. (c) Low expression of transcriptome level was observed in the FAM46C-low cluster which supports a functional cluster mediated by FAM46C. (d) The single cell metastasis model showed the high metastasis contribution of FAM46C low expression group in BMMCs. Thus, the tumor heterogeneity of BMMCs mediated by FAM46C exits in the multiple myeloma.

FAM46C-meditated tumor heterogeneity in BMMCs is functional. The following 3 points support the functional FAM46C-meditated tumor heterogeneity: (a) FAM46C low expression cluster contribute much more than FAM46C high expression cluster. (b) Low expression of FAM46C is closely related to the poorer survival and therapeutic response in multiple myeloma. (c) FAM46C is one of the highest frequently mutated genes with many frameshift and nonsense mutations [18, 29]. Thus, FAM46C-meditated tumor heterogeneity in BMMCs is functional.

What is the role of FAM46C in the FAM46C-meditated heterogeneity cluster? In our research, we can uncover the role of FAM46C layer-by-layer. (a) FAM46C-meditated RNA stability is a distinct role of FAM46C in the heterogeneity cluster. Low expression of transcriptome was observed in the BMMCs vs. CPCs comparison (single cell), FAM46C-low cluster vs. FAM46C-high cluster comparison (single cell), FAM46C low group vs. FAM46C high group comparison (population comparison). (b) FAM46C is closely related to the LDH level of multiple myeloma patients. (c) CITED2 is the top 1 down regulated gene in FAM46C-low vs. FAM46C-high group (population comparison). CITED2 is an efficient brake hypoxic response [30]. Low expression of CITED2 will cause the cell into hypoxia survival [30]. Thus, FAM46C-meditated RNA stability and CITED2 hypoxic response may play a role in the FAM46C-meditated tumor heterogeneity.

## METHODS

### Patients and sample preparation

The participants enrolled in this study were 4 EMP+ (EMP, Extramedullary plasmacytoma) and 2 EMP- primarily untreated MM patients at the Peking University Third Hospital and Beijing Chao-Yang Hospital during 2014-10 to 2015-06 (Supplementary Table 2). All the patients were diagnosed according to the 2009 IMWG (International Myeloma Working Group) consensus criteria [31]. This research was approved by the Peking University Third Hospital ethics committee. Informed consent was obtained in this study in accordance with the Declaration of Helsinki.

4 ml peripheral blood and bone marrow were drawn from untreated MM patients. Peripheral blood mononuclear cells (PBMCs) or bone marrow cells (BMCs) for Single cell RNA sequencing were isolated and purified through Ficoll-Paque Plus (GE Healthcare Bio-Sciences, Pittsburgh, PA, USA) and human CD138 MACS Microbeads (Miltenyi Biotec, Germany) according to the manufacturer's instructions. Single cell suspension was then analyzed or sorted by flow cytometry with antibodies (CD38, CD138) (Biolegend, San Diego, CA, USA) through UltraComp beads (eBioscience/ThermoFisher, USA), Aria II SORP flow cytometer (BD Biosciences, San Jose, CA, USA).

### Single cell RNA sequencing and raw data analysis

Single cell libraries were constructed according to Smartseq2 protocol [32] and sequenced on HiSeq 2500 platform (Illumina, San Diego, CA, USA). Then RNA-seq reads were mapped to the human reference (hg19) using TopHat software (version 2.0.9) allowed for at most 2 mismatches and at least 20nt read length [33, 34]. “*accepted_hits.bam” files of TopHat results were kept for the subsequent analysis. RNA expression was calculated by Cufflinks software (version 2.1.1) using the input “*accepted_hits.bam” files of each sample and quantified as Fragments Per Kilobase of transcript per Million mapped reads (FPKM). The annotation file of each transcript was using “Homo_sapiens.GRCh37.70.gtf” file (Ensembl). FPKM value larger than 1 at least in one cell were taken as stably expressed transcripts [35]. The cells with > 1E5 mapping read and > 1E3 genes numbers were selected. 190 single cells were used for final data analysis after filtering (Supplementary Table 2).

### Single cell RNA sequencing analysis

Immunoglobulin genes of 6 patients were used for One-way analysis of variance. The *P*-value < 1E-6 was defined as specifically expressed immunoglobulin genes in each patient at the single cell transcriptome level.

The transcriptome of clonal circulating plasma cells (CPCs) versus myeloma cells in bone marrow (BMMCs) in P17 and P20 patient were compared using unpaired *t* test. The different expressed genes (DEGs) were defined using the criterion: (a) *P* < 0.05 in unpaired *t* test; (b) foldchange (FC, log2) > 1 or < −1; (c) The criterion of (a and b) should be meet in both P17 and P20 patient. These DEGs may be related to extramedullary metastasis in multiple myeloma.

Unsupervised clustering of BMMCs single cells was done using the DEGs and immunoglobulin genes related genes. Silhouette method was used for cluster selecting. Average silhouette width was calculated for one to ten cluster. The cluster number with the biggest average silhouette width was chosen.

The different expressed genes were used for the whole transcriptome level comparison. *P* < 0.05 in unpaired *t* test and foldchange (FC, log2) > 1 or < −1 was used to defined the different expressed genes if no special designation. For C1 cluster and C2 cluster of P14 BMMCs comparison, *P* < 0.0001 in unpaired *t* test and foldchange (FC, log2) > 1 or < −1 was used to defined the different expressed genes.

The model of extramedullary metastasis in EMP^+^ patient P17 and P20 at single cell transcriptome level was conducted and showed using Sankey diagram. The Pearson correlations were calculated between each CPCs and BMMCs. Only the BMMC with the biggest Pearson correlation value was chosen for a certain CPC. And this BMMC was defined as a possible contributor for a certain CPC. So, a BMMC can contribute 0, 1 or more CPCs. The metastasis rate means a BMMC contributes to how many CPCs. The metastasis rate (M_j_) was calculated using the method in (1).


$$\;\;\;\;\;{\text{M}}_{\text{j}}={\text{C}}_{\text{j}}/{\text{C}}_{p}\;\;\;\;\;\;\;\left(\text{1}\right)$$

Where M_j_ indicates the metastasis rate for the jth BMMC.

Where C_j_ indicates the number of CPC contributed with the jth BMMC.

Where C_p_ indicates the number of CPC analyzed for the certain patient. 30 CPCs for P17 patient and 47 CPCs for P20 patient.

### Gene expression profiling analysis in 2280 multiple myeloma samples

To further analysis the gene expression profiling of multiple myeloma and the relationship between FAM46C expression and clinical characteristics of multiple myeloma, we integrated 7 independent datasets including totally 2280 samples (2072 patients) from the NCBI GEO database. Probeset measures of all the arrays were calculated by RMA (robust multiarray averaging) method. Relative RNA expression value was log-transformed using log2. Data were analyzed with the unpaired *t* test and a *P*-value of < 0.05 was considered to be statistically significantly. Only genes with foldchange (log2) > 1 or < −1 were defined as different expression genes.

GSE24080 with 559 samples (559 patients) were retrieved from the NCBI GEO database. The gene expression array was Affymetrix Human Genome U133 Plus 2.0 Array [36]. The relationship between FAM46C expression and survival (EFS and OS), ISS stages, immunophenotype, FISH 1q21 amplification, molecular subtypes were analyzed.

GSE9782 with 238 samples (238 patients) were retrieved from the NCBI GEO database. The 238 samples were tested by Affymetrix Human Genome U133A Array and Affymetrix Human Genome U133B Array respectively (totally 476 arrays) [37]. FAM46C expression in different therapeutic response with bortezomib or dexamethasone (Dex) was analyzed.

GSE19784 with 308 samples (308 patients) were retrieved from the NCBI GEO database. The 308 samples were tested by Affymetrix Human Genome U133 Plus 2.0 Array [38]. The relationship between FAM46C expression and molecular subtypes were analyzed.

GSE39754 with 136 samples (136 patients) were retrieved from the NCBI GEO database [39]. The 136 samples were tested by Affymetrix Human Exon 1.0 ST Array. FAM46C expression in different therapeutic response with Vincristine, Adriamycin, and Dexamethasone (VAD) and Autologous Stem Cell Transplant (ASCT) were analyzed.

GSE82307 with 66 samples (33 patients) were retrieved from the NCBI GEO database [40]. The 66 samples were tested by Affymetrix Human Genome U133 Plus 2.0 Array. FAM46C expression in presentation (baseline) and relapse paired samples in GSE82307 dataset (totally 33 paired samples) were analyzed. All samples except two were proceeded with at least one autologous hematopoietic stem-cell transplantation (ASCT).

GSE19554 with 36 samples (18 patients) were retrieved from the NCBI GEO database [41]. The 33 samples were tested by Affymetrix Human Genome U133 Plus 2.0 Array. FAM46C expression in baseline and pre-1^st^ (after chemotherapy; pre-1^st^ bone marrow transplant) paired samples in GSE19554 dataset (totally 18 paired samples) were analyzed.

GSE31161 with 937 samples (780 patients) were retrieved from the NCBI GEO database. The 937 samples were tested by Affymetrix Human Genome U133 Plus 2.0 Array [42, 43]. FAM46C expression in baseline and relapse samples with TT2 (autologous hematopoietic stem-cell transplantation and thalidomide therapy) and TT3 (incorporating bortezomib up-front into a tandem transplant regimen) therapy (totally 937 samples) were analyzed.

### Western blot analysis

The best FAM46C-siRNA was selected based on the results of western blot. siRNA was purchased from RiboBio (China). Antibody against FAM46C was purchased from Abcam (UK) and were used at a dilution of 1:1000.

### Detection of H929 cell proliferation

The proliferation ability of H929 cells was examined by CCK8 assay. H929 cells of different groups were seeded into 96-well plates, and the optical density (OD) value at 450 nm was determined at gradient times (48 h, 72 h, 96 h and 120 h) via using an automatic microplate reader.

### Statistical analysis

R software v3.1.3 (ggplot2, ggpubr package) was used in this study. A statistical significance level of *P* < 0.05 was used.

## Supplementary Materials

Supplementary Figures

Supplementary Tables
