# Supplementary material for: FAM46C-mediated tumor heterogeneity predicts extramedullary metastasis and poorer survival in multiple myeloma
Source: Aging (Albany NY). 2023 May 6;15(9):3644–77. doi: 10.18632/aging.204697 (PMC10449297; doi:10.18632/aging.204697)
Supplement: Supplementary Figures [file aging-15-204697-s001.pdf]

SUPPLEMENTARY FIGURES

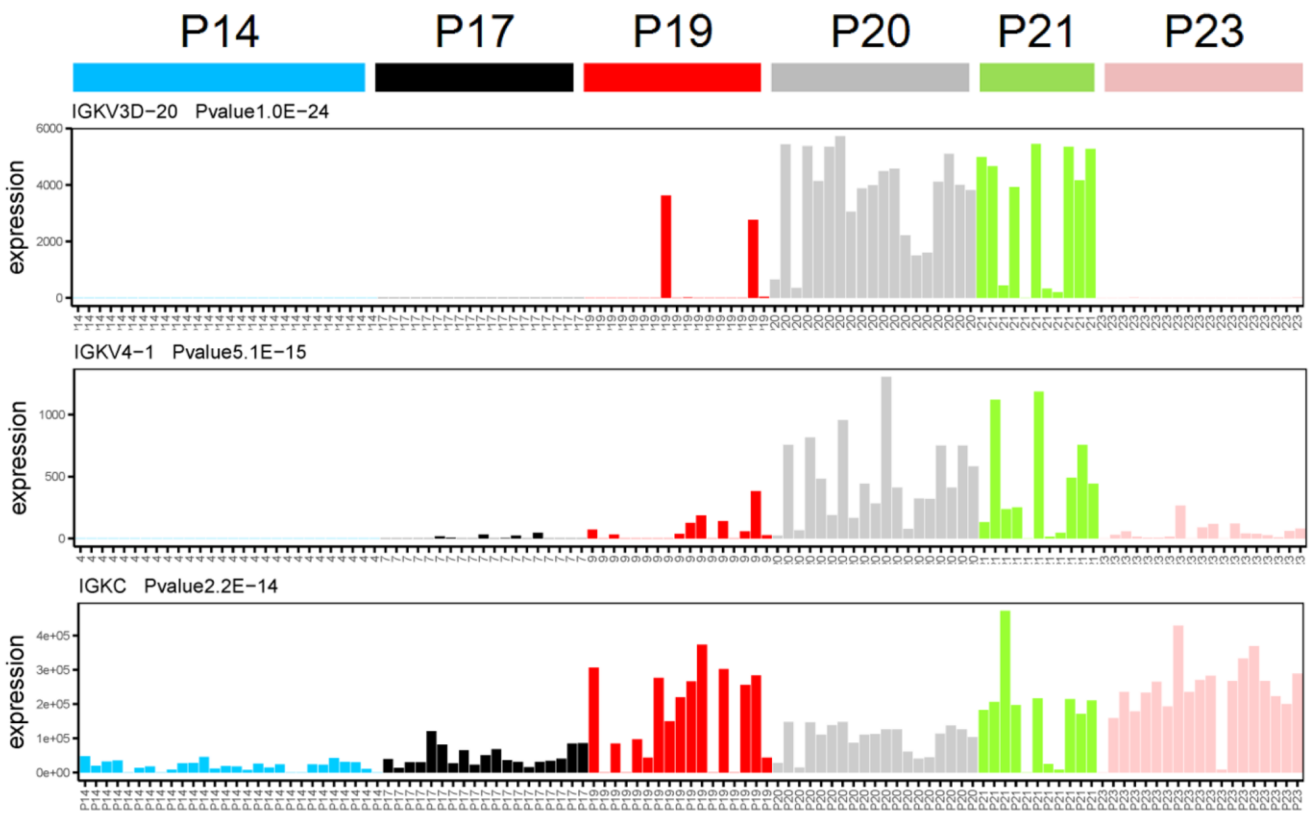

Supplementary Figure 1. Immunoglobulin genes specifically expressed in two to more patients at the single cell transcriptome level. One-way analysis of variance, the gene name and *P*-value were shown in top left corner of each figure.

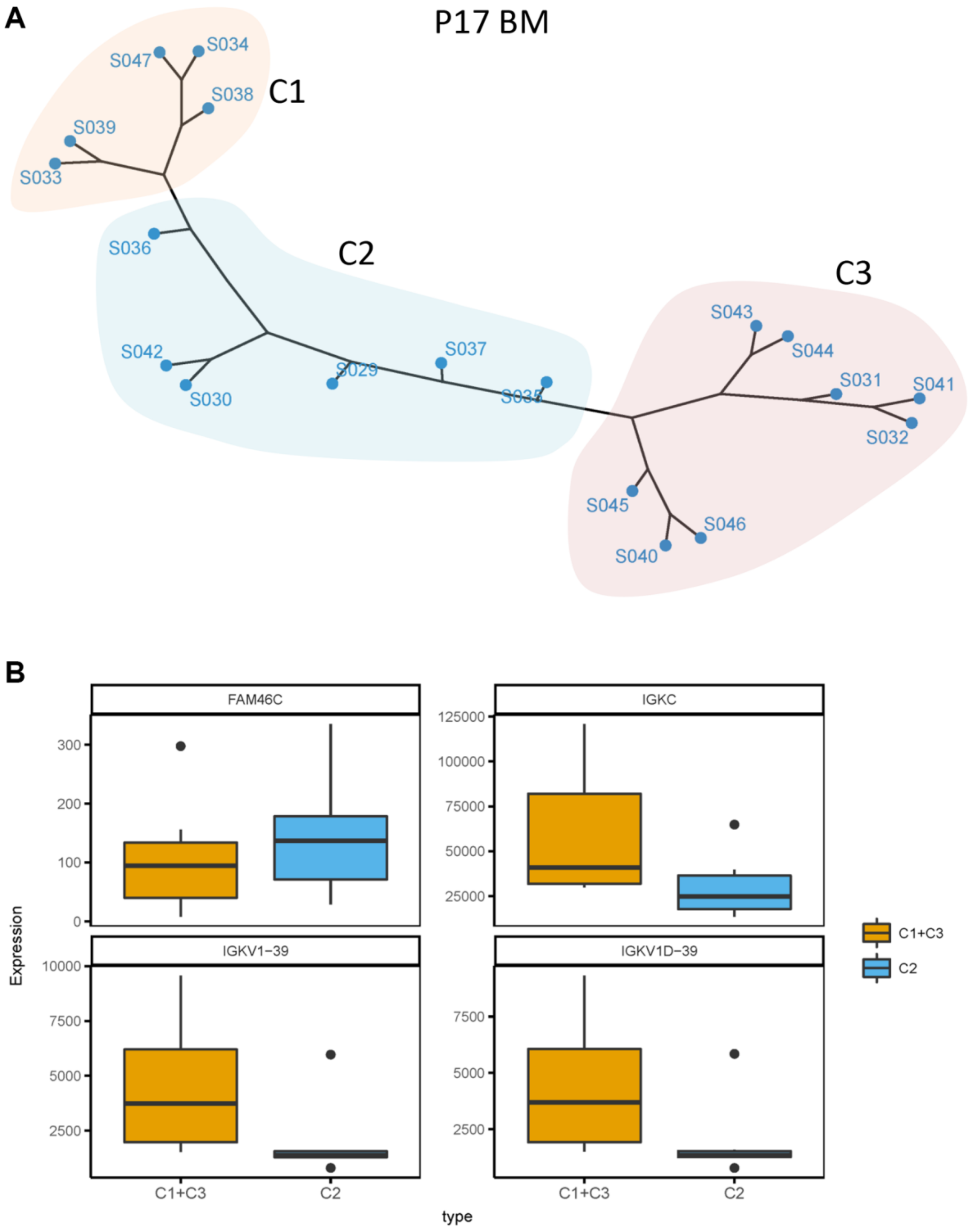

Supplementary Figure 2. Unsupervised clustering of BMMCs of patient P17. (**A**) Unsupervised clustering of BMMCs of patient P17 at the single cell transcriptome level. One point means one single cell of P17 BMMCs. C1, C2, C3 means cluster 1, cluster 2 and cluster 3, showed by 3 different colors. (**B**) FAM46C and immunoglobulin genes trend to differently express in between C1 + C2 and C3. $P < 0.05$, unpaired *t* test, two sided.

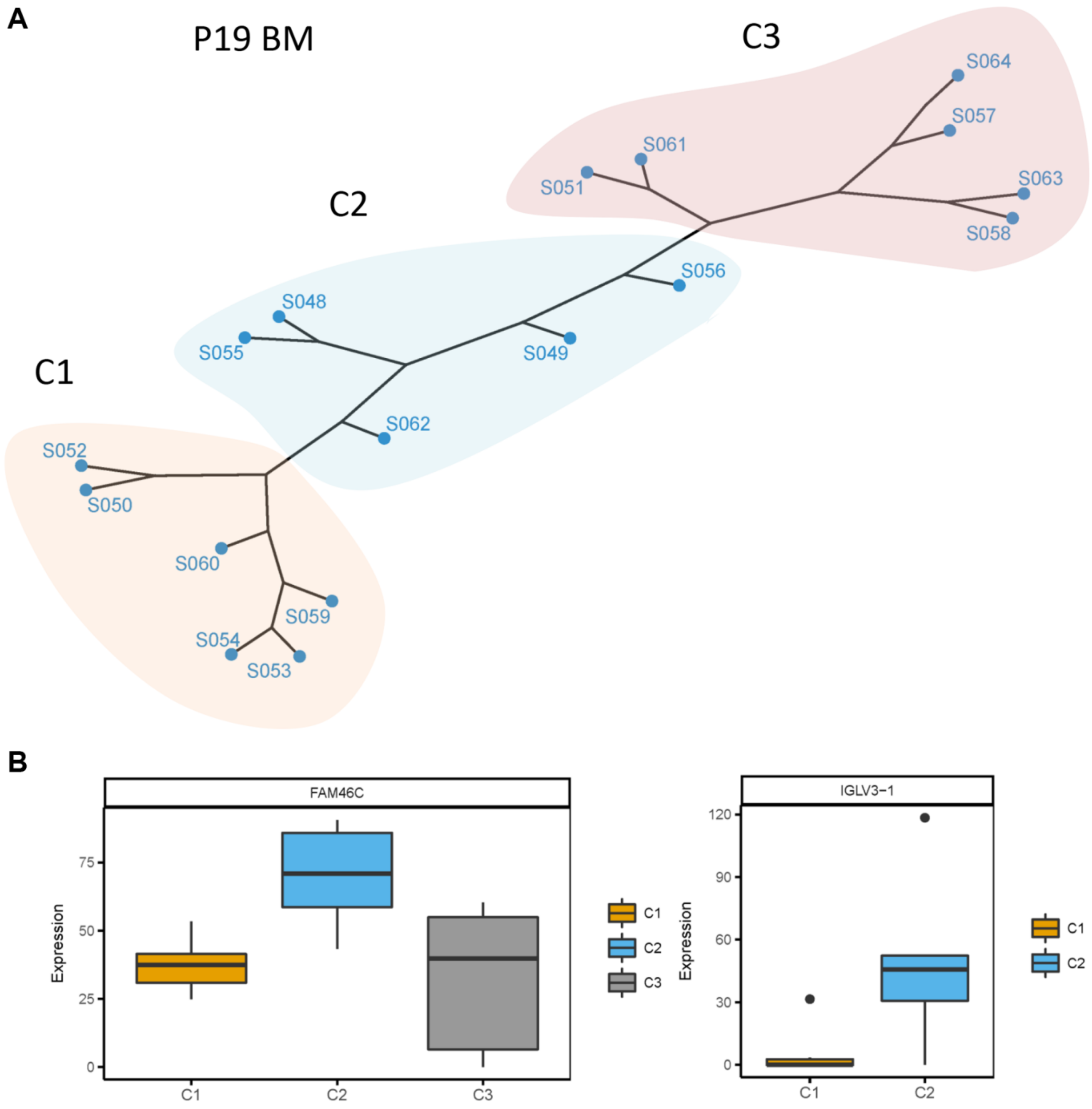

Supplementary Figure 3. Unsupervised clustering of BMMCs of patient P19. (**A**) Unsupervised clustering of BMMCs of patient P19 at the single cell transcriptome level. One point means one single cell of P19 BMMCs. C1, C2, C3 means cluster 1, cluster 2 and cluster 3, showed by 3 different colors. (**B**) FAM46C and immunoglobulin genes were differently expressed in between C1, C2 and C3. $P < 0.05$, unpaired *t* test, two sided.

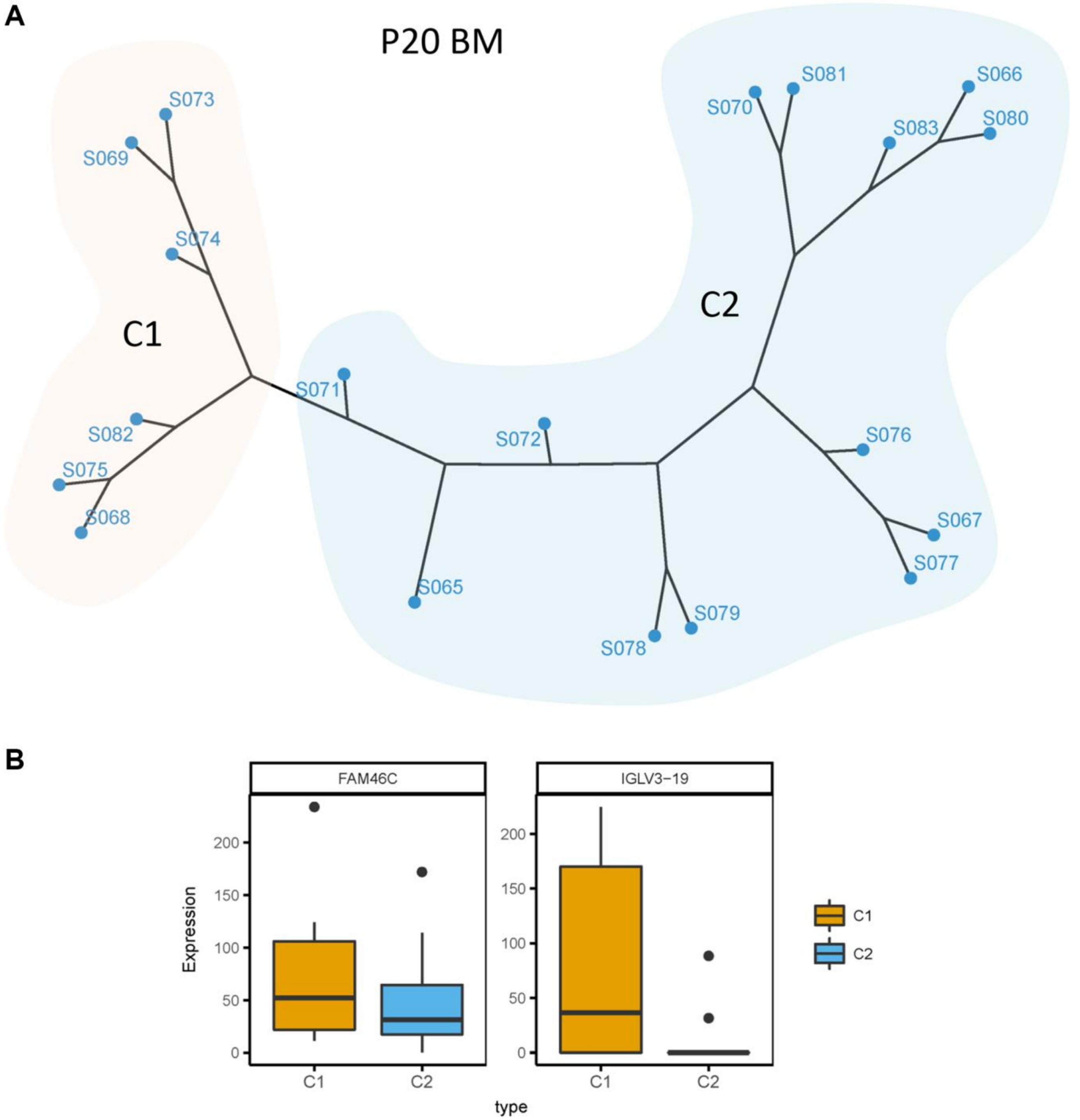

Supplementary Figure 4. Unsupervised clustering of BMMCs of patient P20. (**A**) Unsupervised clustering of BMMCs of patient P20 at the single cell transcriptome level. One point means one single cell of P20 BMMCs. C1, C2 means cluster 1 and cluster 2, showed by 2 different colors. (**B**) FAM46C and immunoglobulin trend to differently express in between C1 and C2. $P < 0.05$, unpaired t test, two sided.

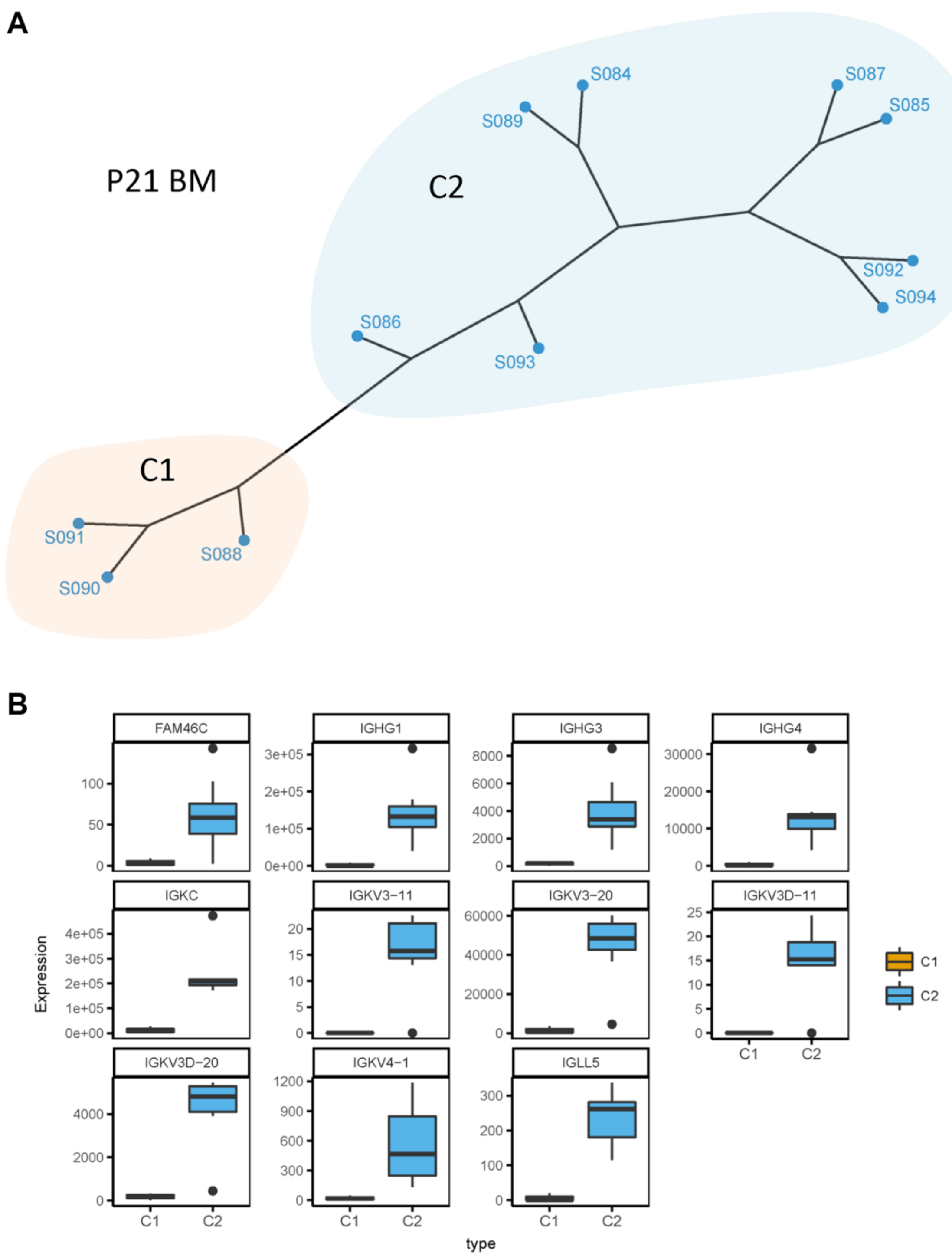

Supplementary Figure 5. Unsupervised clustering of BMMCs of patient P21. (**A**) Unsupervised clustering of BMMCs of patient P21 at the single cell transcriptome level. One point means one single cell of P21 BMMCs. C1, C2 means cluster 1 and cluster 2, showed by 2 different colors. (**B**) FAM46C and immunoglobulin were differently expressed in between C1 and C2. $P < 0.05$, unpaired t test, two sided.

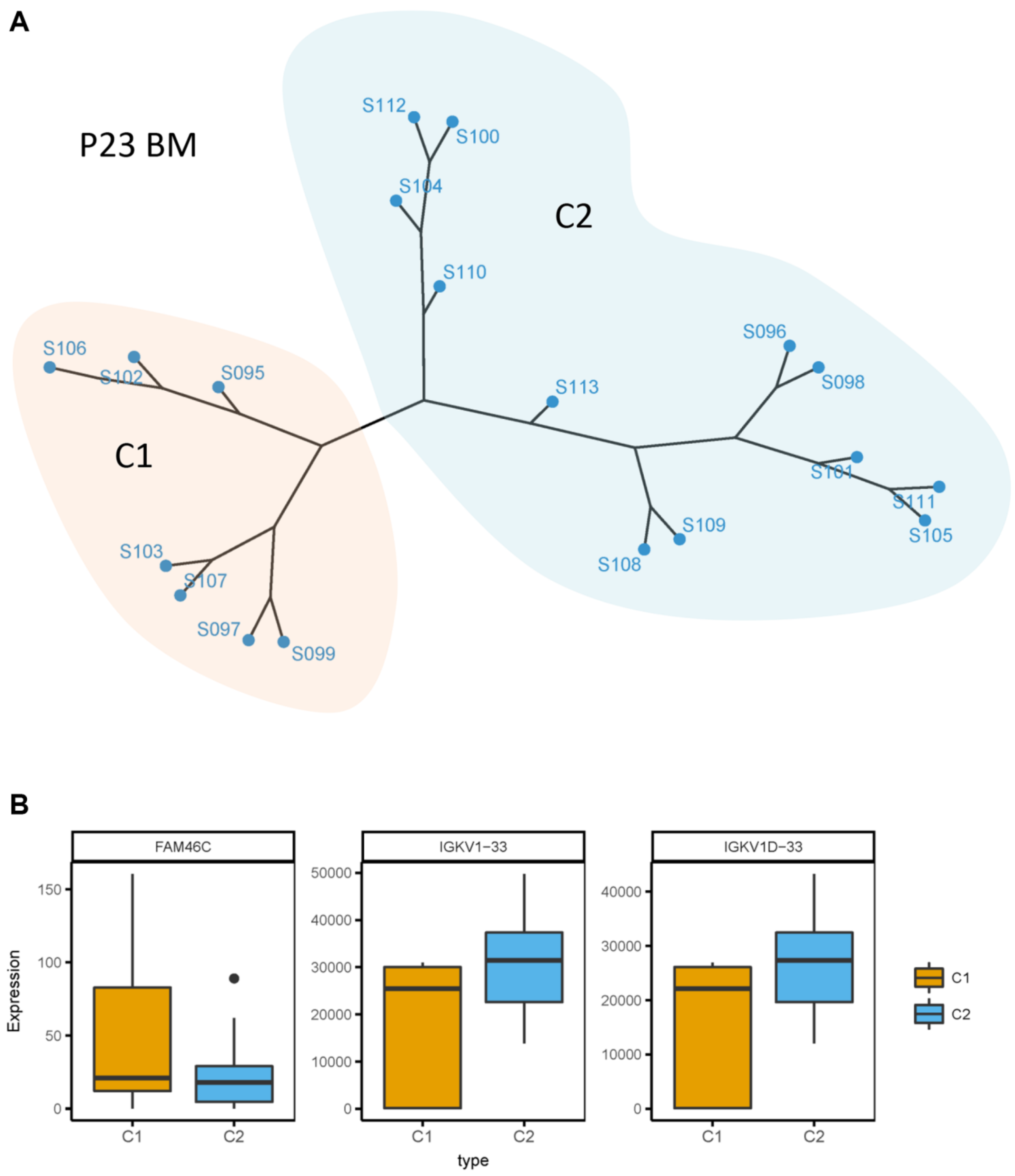

Supplementary Figure 6. Unsupervised clustering of BMMCs of patient P23. (**A**) Unsupervised clustering of BMMCs of patient P23 at the single cell transcriptome level. One point means one single cell of P23 BMMCs. C1, C2 means cluster 1 and cluster 2, showed by 2 different colors. (**B**) FAM46C and immunoglobulin trend to differently express in between C1 and C2. $P < 0.05$, unpaired t test, two sided.

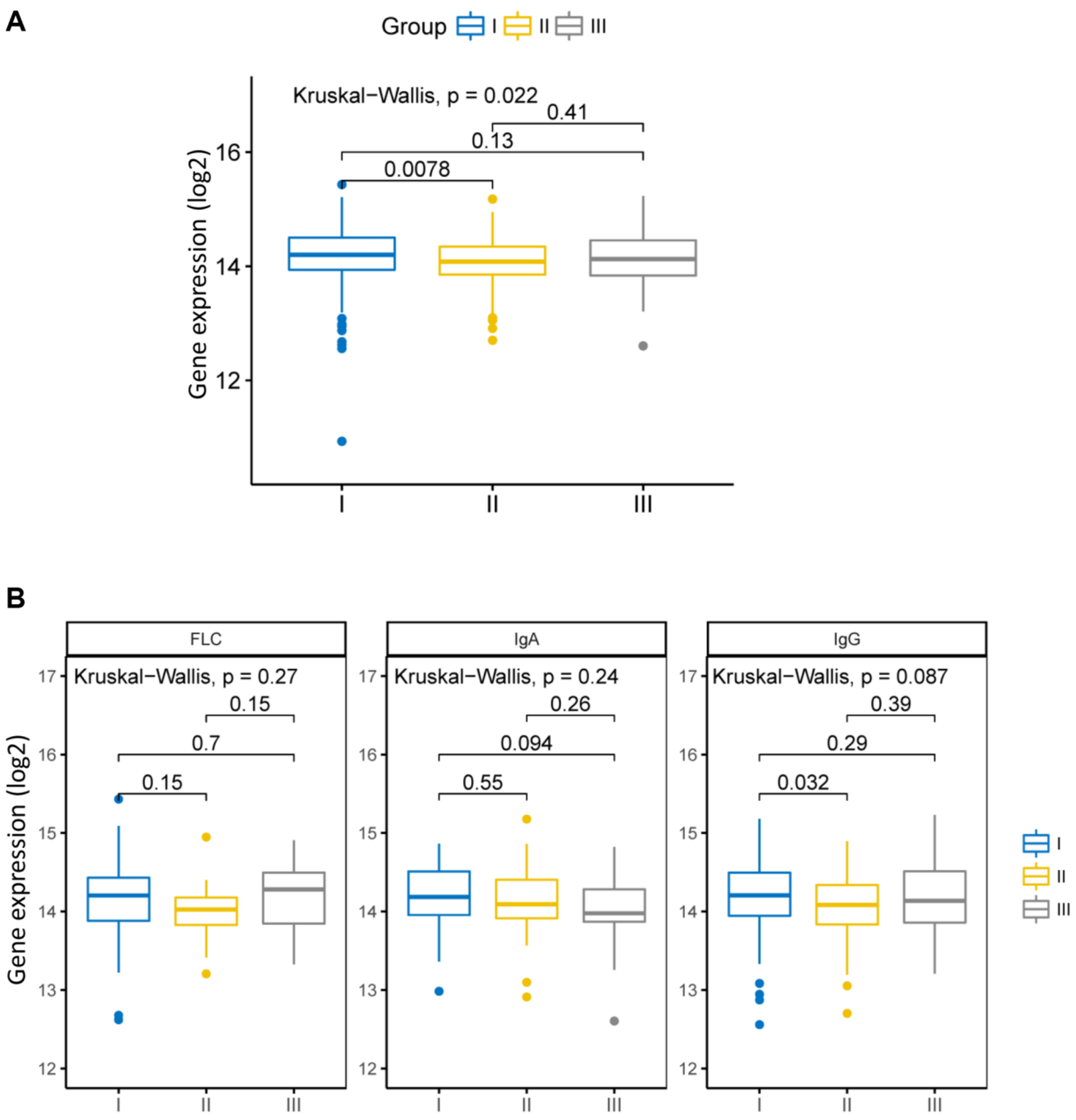

Supplementary Figure 7. FAM46C expression in different ISS stage of 599 multiple myeloma patients. (**A**) FAM46C expression in different ISS stage of 599 multiple myeloma patients. Kruskal-Wallis test. (**B**) FAM46C expression in different ISS stage of FLC, IgG, IgA immunophenotype in 599 multiple myeloma patients. Abbreviation: FLC: free light chain. Kruskal-Wallis test.

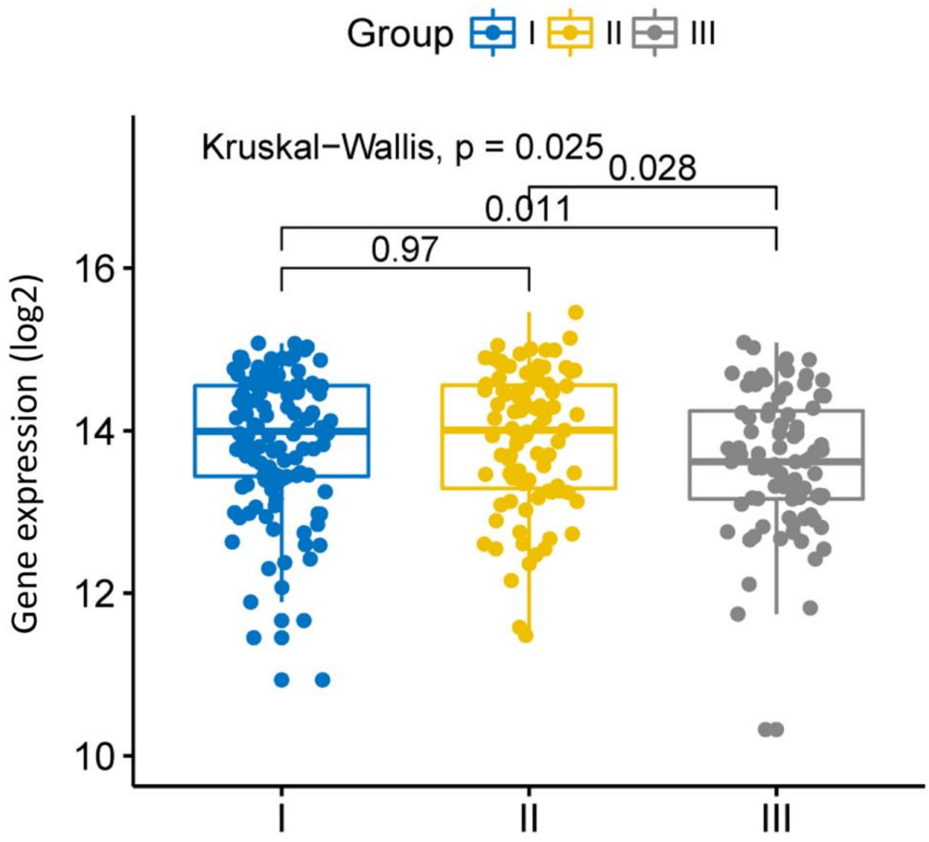

Supplementary Figure 8. FAM46C expression in different ISS stage of another dataset 293 multiple myeloma patients. FAM46C expression in different ISS stage of another dataset (GSE19784) including 293 multiple myeloma patients. Kruskal-Wallis test.

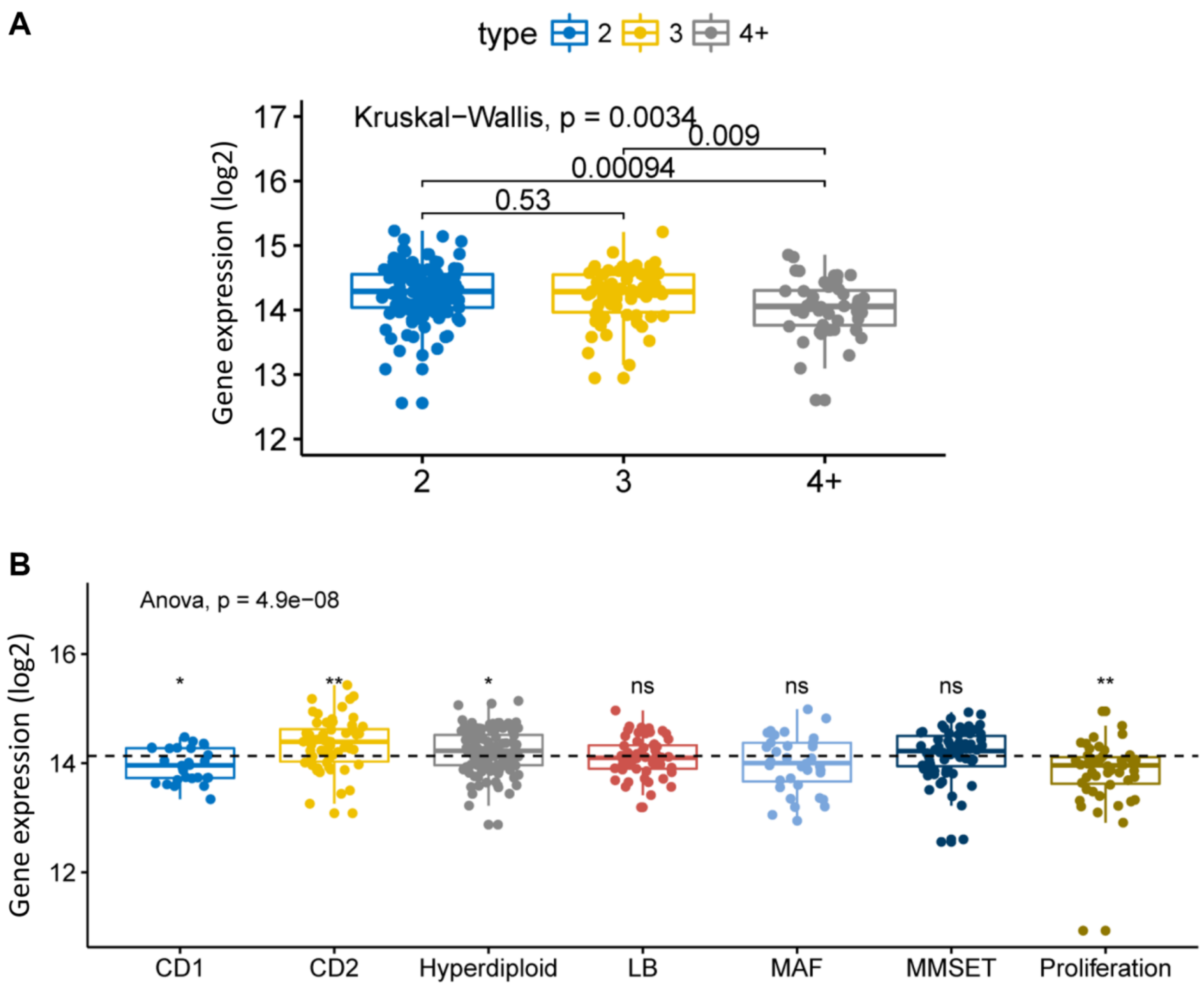

Supplementary Figure 9. FAM46C expression in different molecular subtypes in multiple myeloma patients. (**A**) FAM46C expression in different FISH 1q21 amplification in multiple myeloma patients. Kruskal-Wallis test. (**B**) FAM46C expression in different molecular subtypes in multiple myeloma patients. Kruskal-Wallis test. Symbols indicating statistical significance: ns, $p > 0.05$; $^{*}p \leq 0.05$; $^{**}p \leq 0.01$; $^{***}p \leq 0.001$; $^{****}p \leq 0.0001$. ANOVA (Analysis of variance) test. The mean of the whole dataset was used as reference group. Each of the group levels were compared to the reference group. Horizontal dotted line was added at base mean.

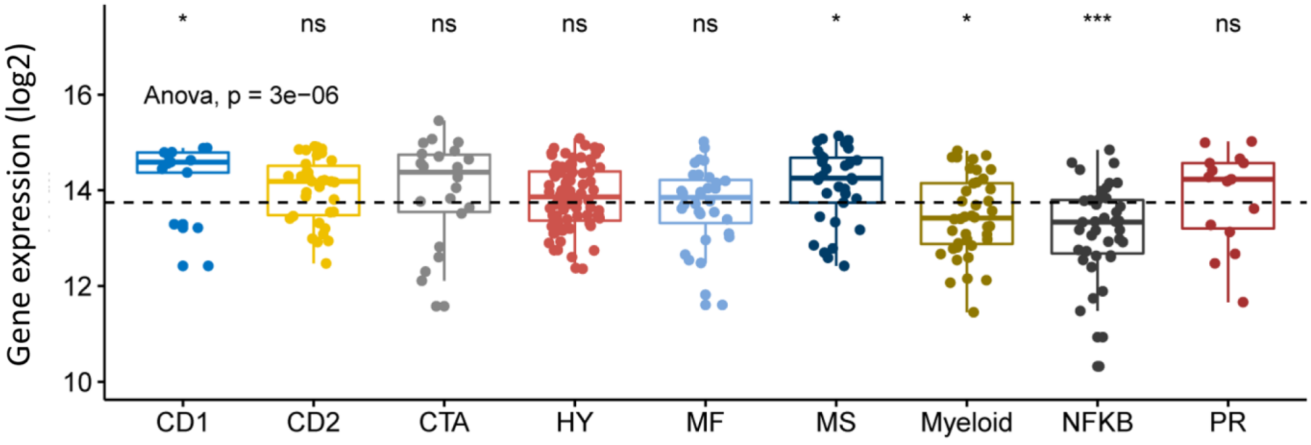

Supplementary Figure 10. FAM46C expression in different molecular subtypes in multiple myeloma patients. FAM46C expression in different molecular subtypes in 308 multiple myeloma patients in another dataset (GSE19784). Kruskal-Wallis test. Symbols indicating statistical significance: ns, $p > 0.05$; $^{*}p \le 0.05$; $^{**}p \le 0.01$; $^{***}p \le 0.001$; $^{****}p \le 0.0001$. ANOVA (Analysis of variance) test. The mean of the whole dataset was used as reference group. Each of the group levels were compared to the reference group. Horizontal dotted line was added at base mean.

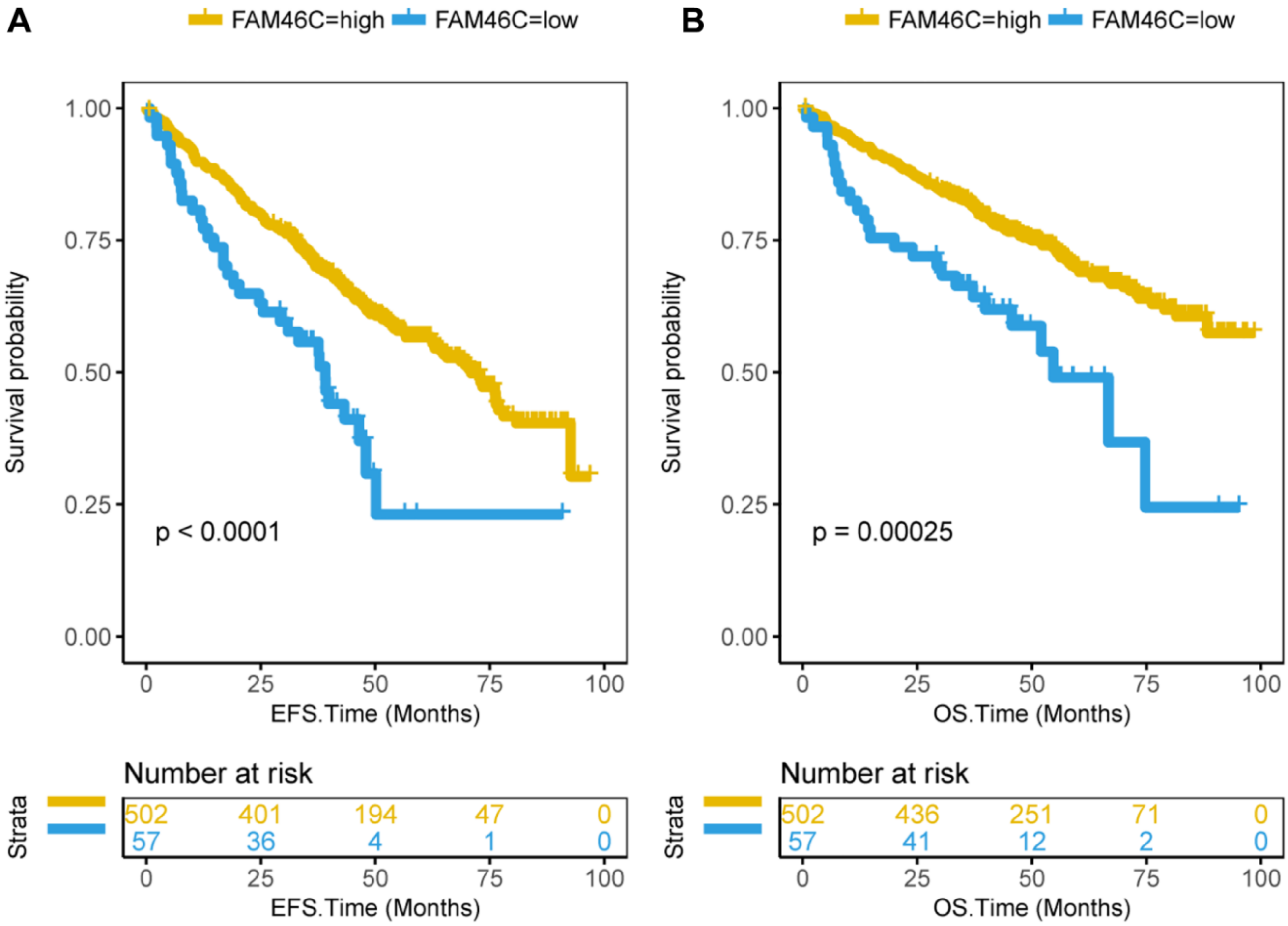

Supplementary Figure 11. The lower expression of FAM46C predicts poorer survival in multiple myeloma. (**A**) Kaplan-Meier curves for EPS of 559 multiple myeloma patients of FAM46C expression. The log-rank test was used to compare Kaplan–Meier curves. (**B**) Kaplan-Meier curves for OS of 559 multiple myeloma patients of FAM46C expression. The log-rank test was used to compare Kaplan–Meier curves.

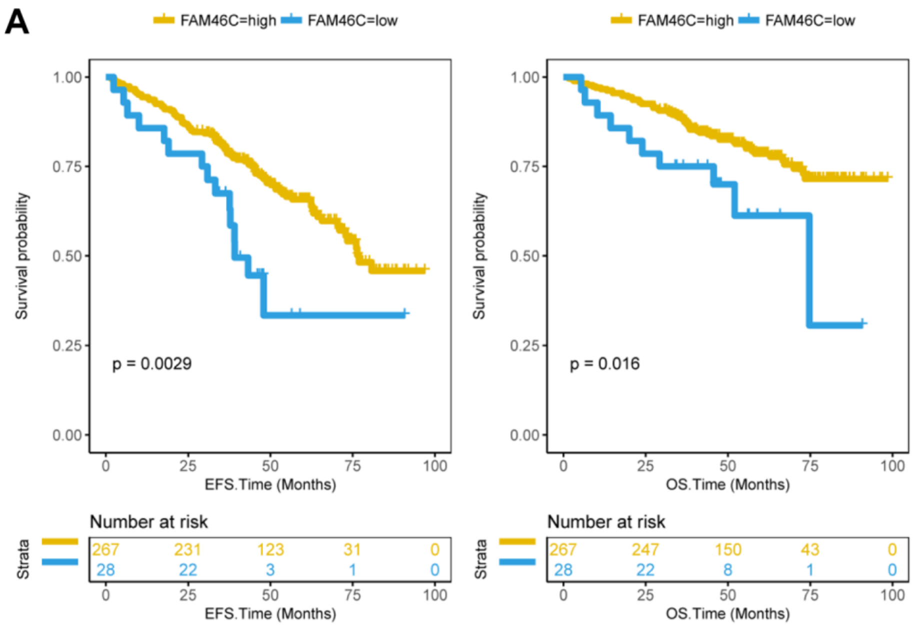

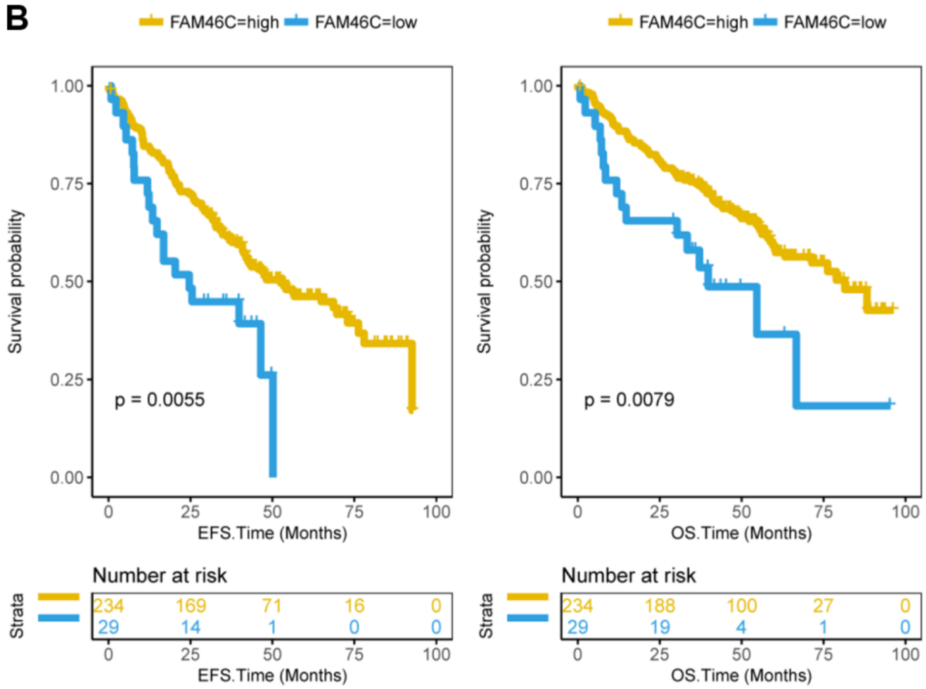

Supplementary Figure 12. The lower expression of FAM46C predicts poorer survival in different ISS stage in multiple myeloma. (**A**) Left plot, Kaplan-Meier curves for EPS of 295 ISS stage I multiple myeloma patients of FAM46C expression. The log-rank test was used to compare Kaplan–Meier curves. Right plot, Kaplan-Meier curves for OS of 295 ISS stage I multiple myeloma patients of FAM46C expression. The log-rank test was used to compare Kaplan–Meier curves. (**B**) Left plot, Kaplan-Meier curves for EPS of 263 ISS stage II and III multiple myeloma patients of FAM46C expression. The log-rank test was used to compare Kaplan–Meier curves. Right plot, Kaplan-Meier curves for OS of 263 ISS stage II and III multiple myeloma patients of FAM46C expression. The log-rank test was used to compare Kaplan–Meier curves.

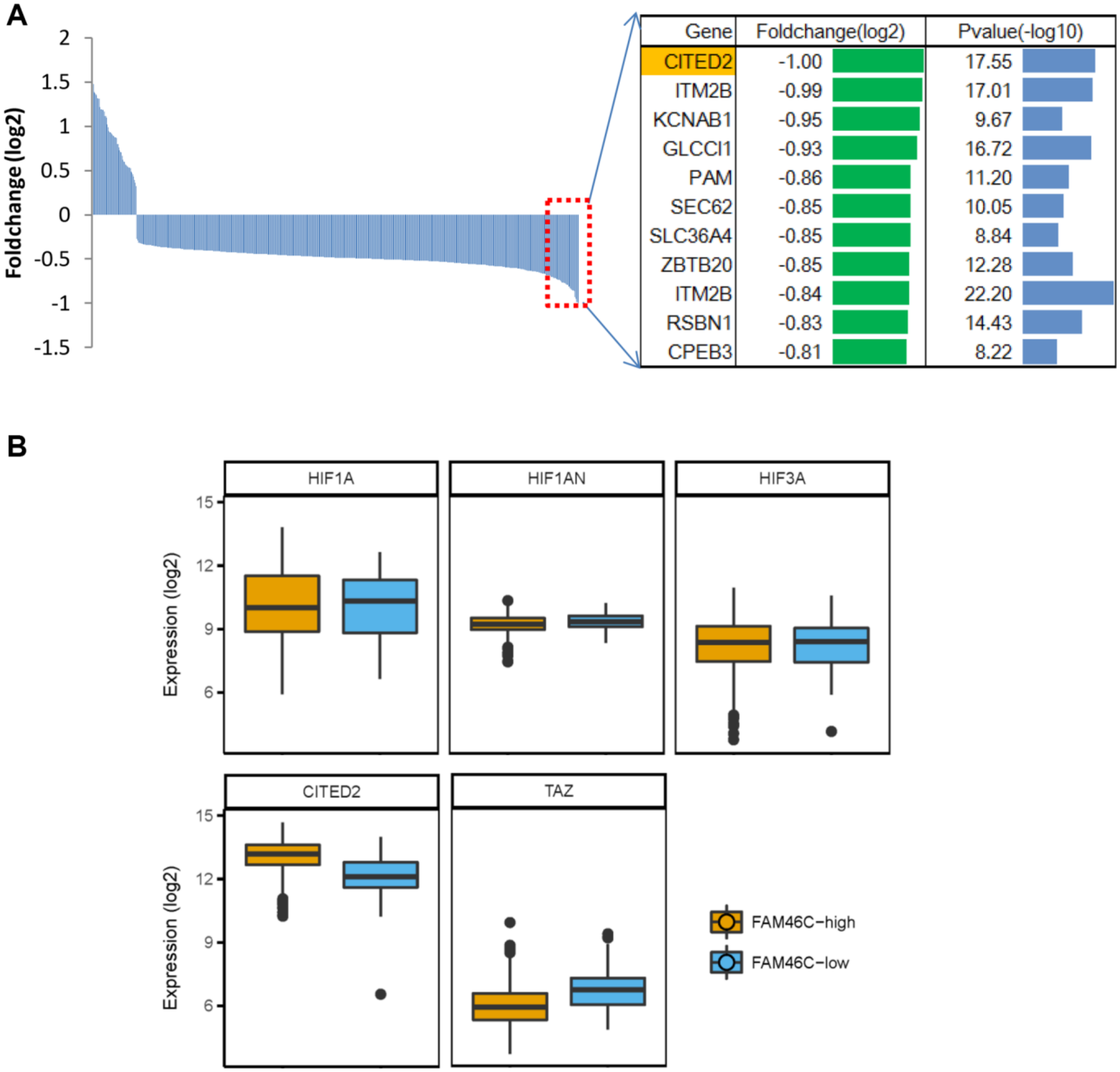

Supplementary Figure 13. The different expression genes in FAM46C-low vs. FAM46C-high group of 559 multiple myelomas. (**A**) All the different expression genes (left) and top 11 (right) in FAM46C-low vs. FAM46C-high group of 559 multiple myelomas. (**B**) Hypoxia related genes expression in FAM46C-low vs. FAM46C-high group of 559 multiple myelomas. Unpaired *t* test, two sided.

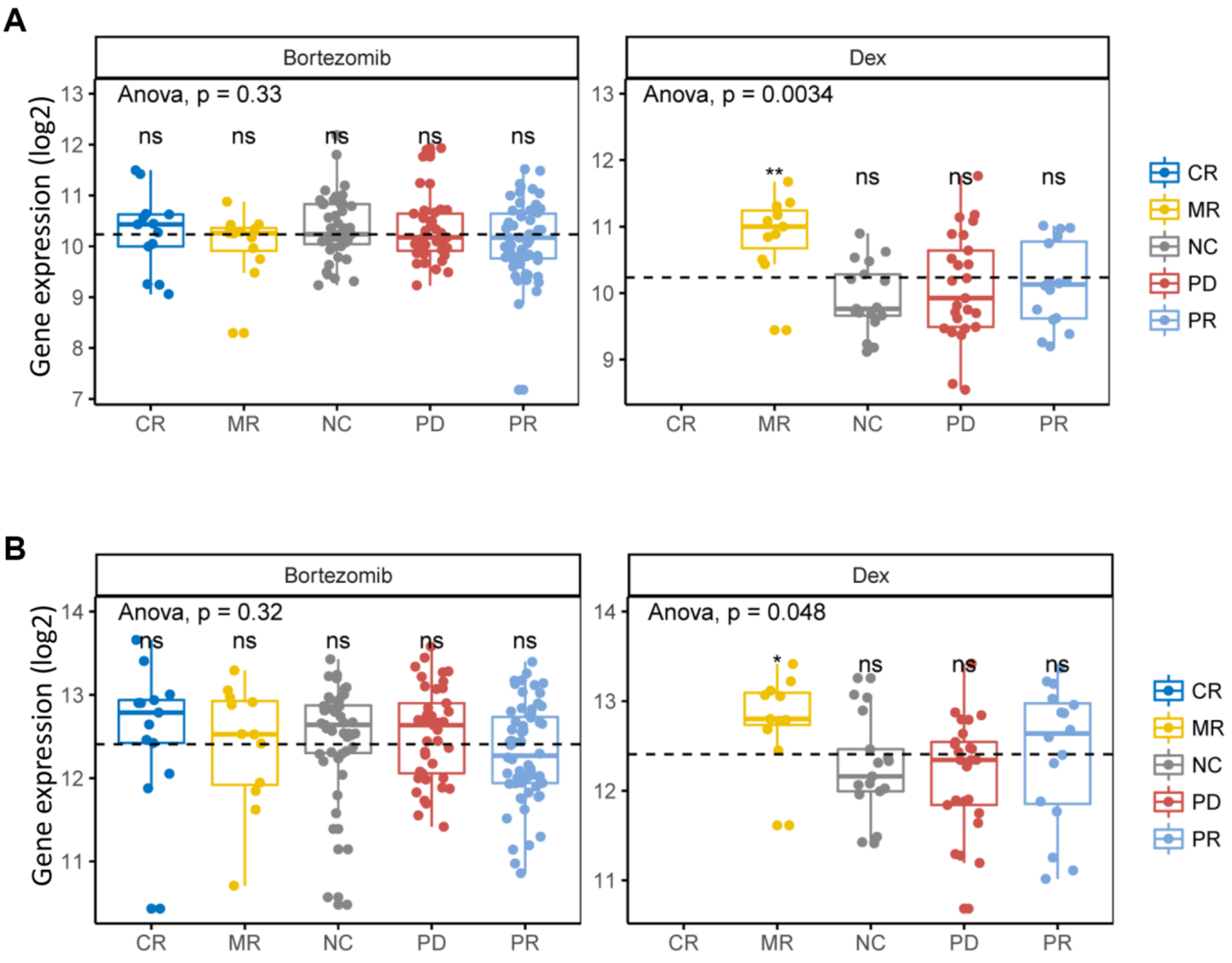

Supplementary Figure 14. FAM46C expression in different therapeutic response with bortezomib or dexamethasone (Dex). (**A**) FAM46C expression in different therapeutic response with bortezomib in 238 multiple myeloma patients (GSE9782) with [HG-U133A] Affymetrix Human Genome U133A Array. (**B**) FAM46C expression in different therapeutic response with bortezomib in 238 multiple myeloma patients (GSE9782) with [HG-U133B] Affymetrix Human Genome U133B Array. Treatment responses: Abbreviations: CR: Complete Response; PR: Partial Response; NC: No Response; PD: Progressive disease; MR: Mini Response. Symbols indicating statistical significance: ns, $p > 0.05$; $^{*}p \leq 0.05$; $^{**}p \leq 0.01$; $^{***}p \leq 0.001$; $^{****}p \leq 0.0001$. ANOVA (Analysis of variance) test. The mean of the whole dataset was used as reference group. Each of the group levels were compared to the reference group. Horizontal dotted line was added at base mean.

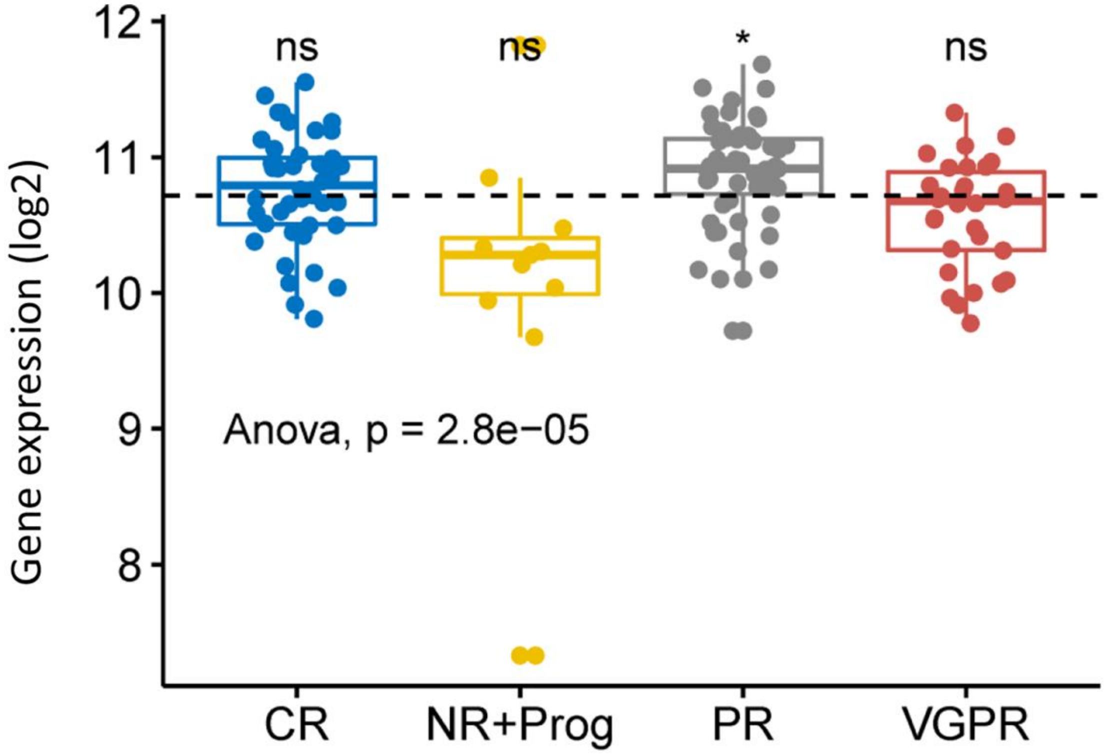

Supplementary Figure 15. FAM46C expression in different therapeutic response. FAM46C expression in different therapeutic response in another dataset (GSE39754) of 136 multiple myeloma patients with Vincristine, Adriamycin, and Dexamethasone (VAD) and Autologous Stem Cell Transplant (ASCT). Treatment responses: Abbreviations: CR: Complete Response; VGPR: Very Good Partial Response; PR: Partial Response; NR: No Response; Prog: Progressive disease. Symbols indicating statistical significance: ns, $p > 0.05$; $^{*}p \leq 0.05$; $^{**}p \leq 0.01$; $^{***}p \leq 0.001$; $^{****}p \leq 0.0001$. ANOVA (Analysis of variance) test. The mean of the whole dataset was used as reference group. Each of the group levels were compared to the reference group. Horizontal dotted line was added at base mean.

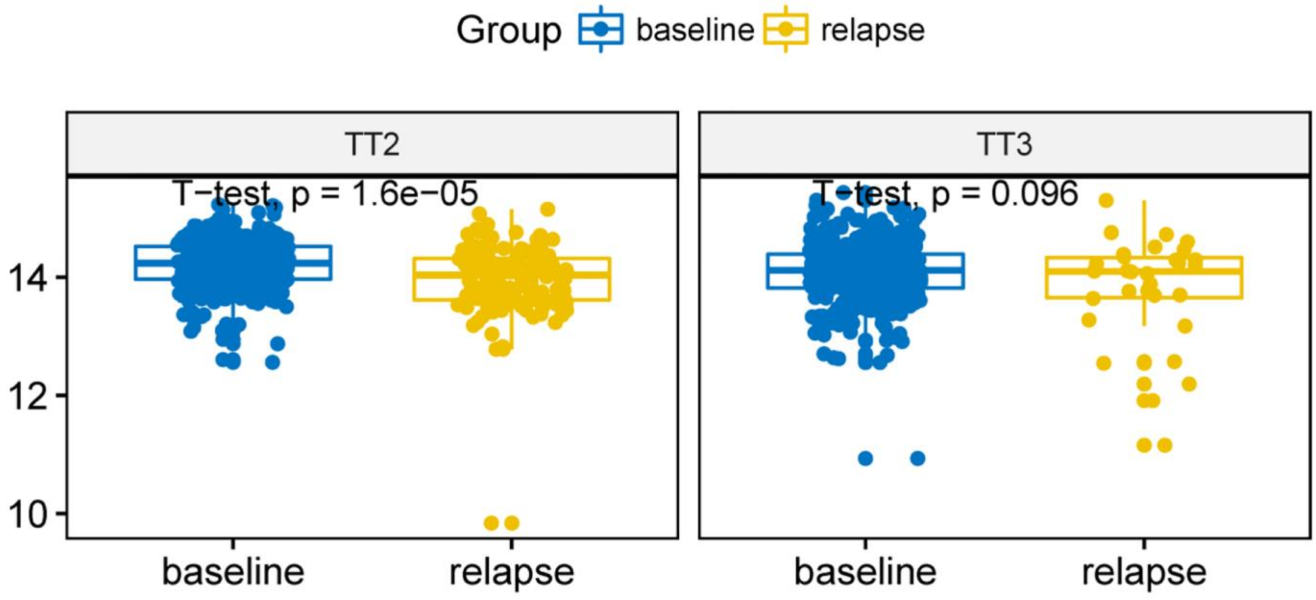

Supplementary Figure 16. FAM46C expression in baseline and relapse samples. FAM46C expression in baseline and relapse samples in GSE31161 dataset with TT2 (left, autologous hematopoietic stem-cell transplantation and thalidomide therapy) and TT3 (right, incorporating bortezomib up-front into a tandem transplant regimen) therapy (totally 937 samples). Unpaired *t* test, two sided.

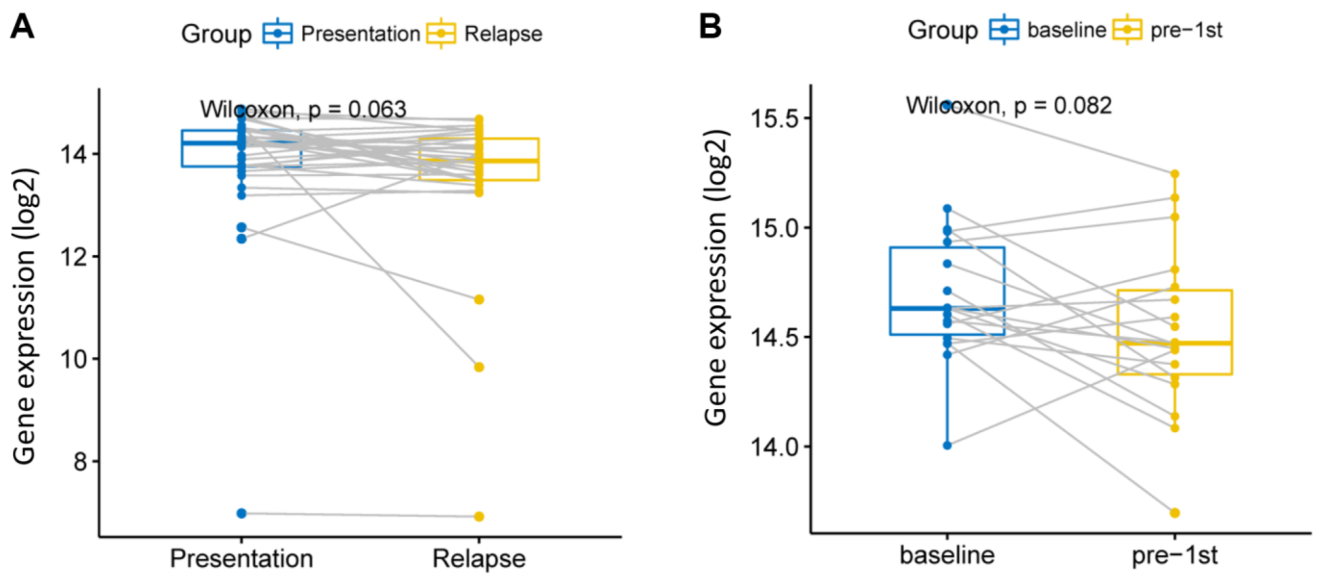

Supplementary Figure 17. FAM46C expression in paired samples of before and after therapy. (**A**) FAM46C expression in presentation (baseline) and relapse paired samples in GSE82307 dataset (totally 33 paired samples). All samples except two proceeded with at least one autologous hematopoietic stem-cell transplantation (ASCT). (**B**) FAM46C expression in baseline and pre-1st (after chemotherapy; pre-1st bone marrow transplant) paired samples in GSE19554 dataset (totally 18 paired samples). Paired *t* test, two sided.
