# Supplementary material for: FAM46C-mediated tumor heterogeneity predicts extramedullary metastasis and poorer survival in multiple myeloma
Source: Aging (Albany NY). 2023 May 6;15(9):3644–77. doi: 10.18632/aging.204697 (PMC10449297; doi:10.18632/aging.204697)
Supplement: Supplementary Tables [file aging-15-204697-s002.pdf]

SUPPLEMENTARY TABLES

Supplementary Table 1. 95 significantly differently expressed genes were found between BMMCs and cPCs in both P17 and P20 patients.

	PBMC vs. BM	PBMC vs. BM	PBMC vs. BM	PBMC vs. BM
**ID**	**P17**	**P17**	**P20**	**P20**
**Type**	**FC**	***P* value**	**FC**	***P* value**
SHC1	−1.7289	0.015	−5.7546	1.3E-14
GANAB	−1.5774	0.00227	−4.233	2.1E-13
SLAMF7	−2.126	0.00029	−3.4001	7.4E-12
IRF4	−1.2338	0.00861	−5.4166	8.8E-11
MAN2A1	−2.2262	0.00874	−4.7581	3.6E-10
MTRNR2L8	2.95762	0.01872	4.85684	7.9E-08
TXNDC5	−1.0376	0.00371	−2.5755	1.3E-07
BTG2	−1.3812	0.00197	−3.3703	1.7E-07
POU2AF1	−2.1768	7E-05	−3.2444	2.7E-07
FAM46C	−1.8532	9.4E-05	−3.5267	2.9E-07
KCNN3	−2.0226	0.01251	−2.9021	4.3E-07
MZB1	−1.0757	0.01224	−2.5107	7.8E-07
SNORD3A	−1.496	0.04556	−2.8061	1.6E-06
NOMO2	−1.5124	0.03537	−2.546	2.8E-06
ALOX5AP	−3.0225	0.0021	−2.9896	3.2E-06
TGOLN2	−2.8476	0.02116	−3.3939	3.2E-06
ZBED6	−1.9243	0.00747	−3.5722	4E-06
CCR2	−3.2103	0.00144	−3.9043	6E-06
NEK9	−2.6786	0.03075	−2.9977	1.1E-05
FNDC3A	−2.5735	0.04964	−3.6116	1.6E-05
FUT8	−2.2422	0.02726	−2.8357	1.6E-05
BSCL2	−2.0737	0.00369	−2.5232	1.7E-05
VOPP1	−1.7465	0.01159	−2.4817	1.9E-05
SULF2	−2.3643	0.00513	−2.7472	2.2E-05
SLC38A2	−1.8483	0.00016	−2.728	3.4E-05
NOMO3	−2.2734	0.00591	−3.2195	4.8E-05
RAC2	−1.8562	0.02608	−2.2361	5.3E-05
SHMT2	−1.9309	0.03362	−3.4989	7.3E-05
SRCAP	−2.1374	0.0006	−2.4755	8E-05
POLR2A	−2.1201	0.01888	−2.4396	9.1E-05
GPR114	−4.1419	0.01344	−4.2574	0.00032
IARS	−2.2027	0.01749	−2.3509	0.00049
ACIN1	−3.2886	0.00304	−3.6991	0.00051
RNF19A	−1.4986	0.01373	−2.2122	0.00053
SIK3	−2.5912	0.01156	−2.7419	0.00063
FASTKD1	−2.9508	0.03705	−3.7125	0.00073
BCOR	−2.6724	0.00168	−3.8076	0.00081
OSBPL3	−2.9286	0.0306	−4.0268	0.00102
IDE	−2.236	0.00328	−3.4248	0.00142
VAC14	−2.1263	0.03366	−3.975	0.00143
AC004447.2	−2.1411	0.00916	−1.8262	0.00154
E2F2	−4.113	0.00813	−3.0402	0.00197
NBEAL2	−3.0182	0.01234	−2.5015	0.00214
ACTR3C	−2.4381	0.01275	−1.5307	0.00253
KANK1	−1.6197	0.04924	−2.9526	0.00264
HIPK2	−2.1466	0.01184	−2.5798	0.00347
IGHV3-23	7.3474	0.0033	3.19899	0.00416
CTD-2619J13.14	−1.3744	0.00127	−2.4502	0.00424

NSF	−1.9021	0.026	−1.7559	0.00521
XRRA1	−2.1513	0.0472	−2.5204	0.0053
DOCK2	−4.5146	0.00152	−2.3046	0.00542
SLC13A3	−1.324	0.03613	−3.1516	0.00638
ZNF460	−1.5418	0.03251	−2.2619	0.00658
TBRG1	−1.9248	0.04335	−3.4894	0.00663
PDS5A	−2.9344	0.00467	−1.8754	0.00668
ADAR	−1.5924	0.01068	−1.8041	0.00705
BAZ2A	−1.1882	0.04463	−2.2956	0.00721
ATAD3C	−1.6287	0.04862	−1.7795	0.00802
MAP4K1	−1.6358	0.04597	−2.2153	0.00814
LRPAP1	−1.8963	0.0166	−1.6959	0.00826
ZBP1	−1.9287	0.04307	−1.8112	0.00928
ELMO1	−3.7704	0.00702	−2.3356	0.00955
FOS	−1.703	0.01442	−2.5704	0.00989
PDXDC2P	−2.5354	0.02918	−2.7736	0.01023
MMACHC	−1.8819	0.02738	−2.0092	0.0103
SEL1L	−1.6216	0.00489	−1.686	0.01078
CNOT4	−2.5722	0.04389	−1.9608	0.01082
CASP10	−3.3902	0.00267	−2.2017	0.01244
SETDB1	−1.9238	0.02903	−2.3407	0.01256
SMARCA2	−3.2094	0.03214	−2.9755	0.01451
NCOA2	−4.0617	0.04477	−2.2601	0.01523
IGLV1-44	7.57289	0.00405	1.93373	0.01524
ST7	−2.0131	0.04718	−3.5356	0.0156
SLC41A1	−2.8949	0.04532	−2.9416	0.01566
RUNX1	−3.0116	0.0217	−2.1007	0.01606
SPTY2D1	−4.126	0.01245	−2.4695	0.01859
CDC73	−1.2557	0.03782	−1.7525	0.01976
PCNX	−3.4407	0.02546	−3.0223	0.02084
DENND1A	−2.3612	0.0236	−1.7931	0.02089
SLC35A4	−1.8958	0.04615	−1.6721	0.02194
HERC2P2	−3.4307	0.01852	−1.7991	0.02406
AP2B1	−2.1699	0.02866	−1.8198	0.02503
DDR2	−3.1547	0.00888	−4.1288	0.02524
NCAPG2	−2.9299	0.02515	−1.298	0.02529
IGHA2	5.23023	0.0025	4.78814	0.02556
BRPF3	−1.9583	0.04551	−1.6205	0.02569
IGHV3-43	3.59169	0.00423	3.96938	0.02608
FTH1	2.61631	0.00019	1.61329	0.02681
C2orf18	−2.383	0.02543	−2.0605	0.0275
UBAP2	−2.1149	0.00816	−2.0553	0.03014
ANGEL1	−3.4752	0.0181	−2.2153	0.03352
PHIP	−1.5215	0.03552	−1.1834	0.04014
RP11-867G23.4	−1.5004	0.0359	−1.2281	0.04141
R3HDM1	−2.0567	0.04427	−2.0878	0.04221
RP11-325F22.2	−2.5729	0.02075	−1.5841	0.04905

Supplementary Table 2. Sample collection and cell detection.

	Patient	BM collected	BMMC detected	PB collected	cPC detected
EMP$^+$	P14	+	28	+	−
(n = 4)	P17	+	19	+	30
	P20	+	19	+	47
	P23	+	19	N.C.	
EMP$^-$	P19	+	17	+	−
(n = 2)	P21	+	11	N.C.	

Abbreviations: EMP: Extramedullary plasmacytoma; BM: bone marrow; PB: peripheral blood; +: collected or detected; N.C.: not collected; −: not detected. Number in bracket means number of cells sequenced and used for final data analysis.
